# Charge Redistribution Mechanisms in SnSe_2_ Surfaces Exposed to Oxidative and Humid Environments and Their Related Influence on Chemical Sensing

**DOI:** 10.1021/acs.jpclett.0c02616

**Published:** 2020-10-09

**Authors:** Gianluca D’Olimpio, Francesca Genuzio, Tevfik Onur Menteş, Valentina Paolucci, Chia-Nung Kuo, Amjad Al Taleb, Chin Shan Lue, Piero Torelli, Daniel Farías, Andrea Locatelli, Danil W. Boukhvalov, Carlo Cantalini, Antonio Politano

**Affiliations:** † Department of Physical and Chemical Sciences, University of L’Aquila, via Vetoio, 67100 L’Aquila, AQ, Italy; ‡ Elettra-Sincrotrone S.C.p.A., S.S. 14-km 163.5 in AREA Science Park, 34149 Trieste, Italy; § Department of Industrial and Information Engineering and Economics, University of L’Aquila, Via G. Gronchi 18, I-67100 L’Aquila, Italy; ∥ Department of Physics, National Cheng Kung University, 1 Ta-Hsueh Road, 70101 Tainan, Taiwan; ⊥ Departamento de Física de la Materia Condensada, Universidad Autónoma de Madrid, 28049 Madrid, Spain; # Consiglio Nazionale delle Ricerche (CNR)-Istituto Officina dei Materiali (IOM), Laboratorio TASC in Area Science Park S.S. 14 km 163.5, 34149 Trieste, Italy; @ Instituto ‘Nicolás Cabrera’, Universidad Autónoma de Madrid, 28049 Madrid, Spain; ∇ Condensed Matter Physics Center (IFIMAC), Universidad Autónoma de Madrid, 28049 Madrid, Spain; ● College of Science, Institute of Materials Physics and Chemistry, Nanjing Forestry University, Nanjing 210037, P. R. China; ○ Theoretical Physics and Applied Mathematics Department, Ural Federal University, Mira Street 19, 620002 Ekaterinburg, Russia; ■ CNR-IMM Istituto per la Microelettronica e Microsistemi, VIII strada 5, I-95121 Catania, Italy

## Abstract

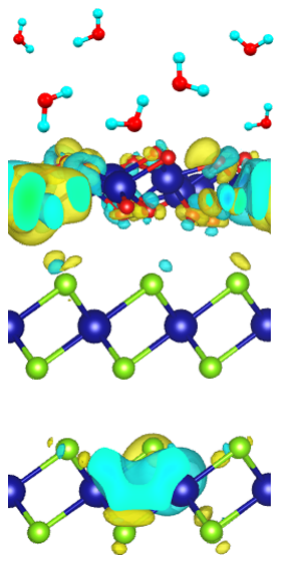

Tin diselenide (SnSe_2_) is a van der Waals semiconductor, which spontaneously forms a subnanometric SnO_2_ skin once exposed to air. Here, by means of surface-science spectroscopies and density functional theory, we have investigated the charge redistribution at the SnO_2_–SnSe_2_ heterojunction in both oxidative and humid environments. Explicitly, we find that the work function of the pristine SnSe_2_ surface increases by 0.23 and 0.40 eV upon exposure to O_2_ and air, respectively, with a charge transfer reaching 0.56 e^–^/SnO_2_ between the underlying SnSe_2_ and the SnO_2_ skin. Remarkably, both pristine SnSe_2_ and defective SnSe_2_ display chemical inertness toward water, in contrast to other metal chalcogenides. Conversely, the SnO_2_–SnSe_2_ interface formed upon surface oxidation is highly reactive toward water, with subsequent implications for SnSe_2_-based devices working in ambient humidity, including chemical sensors. Our findings also imply that recent reports on humidity sensing with SnSe_2_ should be reinterpreted, considering the pivotal role of the oxide skin in the interaction with water molecules.

After the advent of graphene,^1−3^ van der Waals semiconductors are attracting considerable attention, owing to their application capabilities that are often complementary to those of graphene,^4−6^ with the subsequent prospect of novel disruptive technologies in different technological areas.^4,7,8^ This class of materials is characterized by weak van der Waals bonds between layers enabling their cleavage by mechanical^9^ and liquid-phase^10^ exfoliation. Among van der Waals semiconductors, several materials show serious drawbacks, limiting their technological potential. Specifically, MoS_2_ and WS_2_ display intrinsic electron mobility as low as some tens of cm^2^ V^–1^ s^–1^ at 300 K;^11^ black phosphorus rapidly degrades in air due to surface oxidation;^12,13^ GaSe is affected by both environmental and laser-induced degradation;^14,15^ and PdSe_2_^16^ has a limited commercial potential, due to the constantly growing price of Pd ($2000–2400/oz), which nearly doubled from 2019 to 2020.

Tin diselenide (SnSe_2_) is a van der Waals semiconductor with a CdI_2_-type crystal structure,^17^ belonging to the *P*3̅*m*1 space group, with tin (Sn) atoms interweaved between two hexagonally packed atomic layers of selenium (Se) (see the atomic structure in Figure S1a,b).^18,19^ SnSe_2_ shows its high intrinsic electron mobility (462.6 cm^2^ V^–1^ s^–1^ at 300 K^20^) and ultralow thermal conductivity (3.82 W m^–1^ K^–1^^20^). It displays pressure-induced periodic lattice distortion, and moreover, it enables novel device functionalities being a phase change memory material; i.e., its atomic structure can reversibly switch from amorphous to crystalline upon laser heating, with consequent remarkable variations in optical reflectivity. Because of these peculiarities, SnSe_2_ has high application capabilities in numerous fields, including photocatalysis,^21,22^ superconductivity,^23,24^ Li-ion^18,25,26^ and Na-ion^18,26,27^ batteries, photodetection,^28^ saturable absorbers for eye-safe lasers,^29^ and thermoelectricity.^30−32^ Furthermore, SnSe_2_ was used as a co-catalyst for hydrogen evolution reaction.^33^

However, all Sn-based chalcogenides are usually affected by rapid surface degradation with the emergence of tin oxide phases.^34,35^ Additionally, the oxidation of starting element Sn during the synthesis can also influence the transport properties of the resulting crystal. Therefore, technological exploitation of Sn-based chalcogenides remains particularly challenging. Especially, the stability of SnSe_2_-based devices in the ambient atmosphere is related to the chemical reactivity of its surface.

Recently, it has been shown that, though stoichiometric SnSe_2_ shows outstanding chemical stability under ambient conditions, the presence of Se vacancies drastically affects surface chemical reactivity.^36^ The SnSe_2–*x*_ surface is transformed into SnO_2_ skin-terminated SnSe_2_, with the thickness of the SnO_2_ skin estimated to be subnanometric.^36^ Unexpectedly, the self-assembled heterostructure formed by exploiting the natural interaction with air is particularly appropriate for ultrasensitive gas sensing, as demonstrated for NO_2_ and H_2_ with sensitivities of (1.06 ± 0.03) and (0.43 ± 0.02) ppm^–1^.^36^ Remarkably, such sensors are effective under dry air conditions, while previously devised SnSe_2_ sensors used N_2_ as the carrier gas.^37,38^ Moreover, the NO_2_ sensitivity of the SnO_2_–SnSe_2_ heterostructure is significantly higher compared to those of sensors based on other van der Waals semiconductors and their heterostructures.^39,40^

Remarkably, the oxide skin plays a pivotal role in NO_2_ and H_2_ sensing, congruently with the abundant literature on SnO_2_-based sensors.^41−50^ The modulation of resistivity upon gas adsorption is strictly connected to charge distribution in the sensing material, ultimately related to the formation of surface dipoles at the SnO_2_–SnSe_2_ heterojunction arising from local charge redistribution. Thus, to understand the conduction mechanism ruling chemical sensing, it is crucial to shed light on charge redistribution at the SnO_2_–SnSe_2_ heterostructure by measuring work-function changes. Furthermore, sensing experiments in ref (36) were carried out in dry air; thus, stability in a humid environment remains unexplored, although real conditions mandatorily require sensors to work in a changing humidity background^51,52^ (not only humidity sensors^53,54^). Despite the relevance of the influence of the humid environment for practical applications, surprisingly it has been scarcely investigated, although previous reports indicated a decrease in resistance under exposure to a humid atmosphere,^51,52^ which represents an unambiguous fingerprint that H_2_O behaves as a reducing gas in the interaction with the SnO_2_ surface.

In addition, the interaction with water is relevant also for understanding the stability of any other SnSe_2_-based (opto)electronic device^55^ working in ambient humidity, as well as the eventual environmental doping effects in transport properties.^56^ Actually, recently different groups have reported that SnSe_2_ is extremely sensitive to humid environments,^37,38,57^ with the possibility of using it in humidity-sensing devices.

Here, we unveil the surface properties of SnSe_2_ single crystals and their modifications in oxidative and humid environments by means of surface-science experiments and density functional theory (DFT). Definitely, surface oxidation induces an increase in the work function of 0.4 eV, owing to the charge transfer between the substrate and the SnO_2_ skin of 0.56 e^–^ per SnO_2_ unit. As opposed to previous reports,^37,38,57^ the pristine SnSe_2_ surface is inert to water at room temperature, while the SnO_2_–SnSe_2_ heterostructure displays notable sensitivity to humidity.

The presence of the SnO_2_ skin in the SnSe_2_ surface exposed to oxidative environments was ensured by both microscopic evidence from low-energy electron microscopy (LEEM) (Figure S5) and vibrational experiments from high-resolution electron energy loss spectroscopy (HREELS) (Figure S6).

The analysis of the variation of work function ΔΦ probed by LEEM could provide important insights into charge redistribution arising from surface oxidation (Figure 1a), as the total reflectivity threshold in electron backscattering (the MEM–LEEM transition, where MEM stands for mirror electron microscopy) represents a direct measurement of the variation of the surface potential.^58^ Explicitly, we find ΔΦ to be 0.23 eV for the SnSe_2_ surface modified by exposure to 700 L of O_2_ at room temperature, while air exposure for 15 min induces a further shift in the work function, resulting in a total increase of 0.40 eV. The observed value of ΔΦ can be explained by considering the activation of surface dipoles, due to charge transfer at the interface from substrate to adsorbed oxygen atoms. The electronegativity of oxygen makes its adsorption generally associated with a charge transfer from the substrate to the adsorbate layer, with a subsequent increase in the work function.^59^ Considering that the work function of the pristine SnSe_2_ single crystal is ∼4.6 eV,^60^ while that of SnO_2_ is known to be ∼4.9 eV^61^ (although its value can be tuned by reduction reactions^62^), both the sign and the magnitude of the experimental value of ΔΦ are consistent with surface oxidation, involving the formation of a subnanometric SnO_2_ skin. We can infer that previous experimental studies reporting a work function of SnSe_2_ of (5.0 ± 0.1) eV^63,64^ could be affected by surface oxidation, which generates a self-assembled SnO_2_–SnSe_2_ heterostructure with an increased work function. To verify this statement, we calculated ΔΦ for the oxidation of the pristine SnSe_2_ surface, finding a value of 0.52 eV in qualitative agreement with experimental measurements. We also note that, in the air-exposed sample, variations in the *I*–*V* curve associated with electron diffraction from a surface with crystalline order^65^ are suppressed, due to the formation of a disordered surface oxide phase.

**Figure 1:**
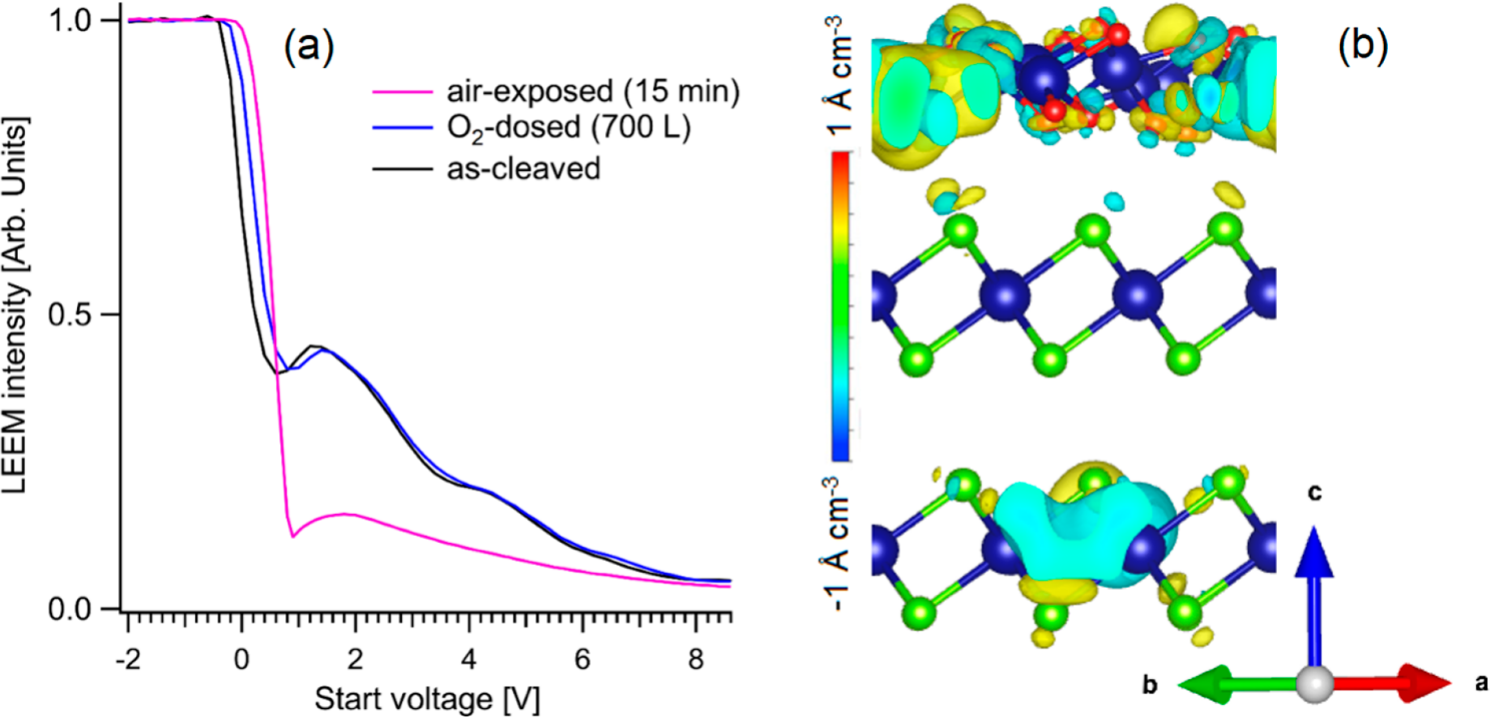
(a) LEEM *I*–*V* curves at the MEM–LEEM transition for the as-cleaved sample (black), after a dose of 700 L of O_2_ (blue), and after air exposure for 15 min (pink). The shift of the MEM–LEEM transition, characterized by the sharp decrease in intensity, indicates an oxidation-induced modification of the surface potential. (b) Changes in charge density after the formation of the interface between the SnSe_2_ substrate and SnO_2_ skin. Sn, Se, and O atoms are represented as dark blue, light green, and red balls, respectively.

Complementary information about the electronic properties of the SnO_2_–SnSe_2_ heterostructure was achieved by comparing the surface excitation spectrum probed by electron energy loss spectroscopy (EELS) with the theoretical density of states (DOS) (section S4 of the Supporting Information). The impact of defects on the DOS is assessed in section S5.

To estimate the amount of charge transfer between the SnSe_2_ substrate and the SnO_2_ skin, we calculated the charge density distribution of (i) one SnO_2_ layer over two layers of SnSe_2_ (to model the SnO_2_–SnSe_2_ heterostructure), (ii) a free-standing SnO_2_ single unit, and (ii) a bilayer of SnSe_2_. Then, we calculated the difference between the charge densities of the whole SnO_2_–SnSe_2_ interface and those one of its components (single SnO_2_ unit and bilayer SnSe_2_). The obtained charge density difference (Figure 1b) illustrates charge redistribution following the formation of the SnO_2_–SnSe_2_ interface. The integration of the charge density difference along the *c* axis provides information regarding the charge transfer between the SnSe_2_ substrate and the SnO_2_ skin. Note that the formation of the SnO_2_–SnSe_2_ interface provides changes in the charge density difference in not only the outermost SnSe_2_ layer but also the subsurface area, namely the second SnSe_2_ layer. Definitely, the charge transfer is estimated to be 0.56 e^–^ per SnO_2_ unit.

While the adsorption of O_2_ with further decomposition is energetically favorable on SnSe_2_ (negative values of Δ*G* and Δ*H*_dec_), as well as on SnSe_1.88_ and SnSe (Table 1), our theoretical model indicates that water does not adsorb on SnSe_2_. The energy cost for water adsorption is decreased in the presence of Se vacancies (SnSe_1.88_) down to ∼3 kJ/mol, although water adsorption (as well as decomposition) remains energetically unfavorable. Similarly, SnSe also shows outstanding chemical inertness toward water.

**Table 1:** Differential Enthalpies (Δ*H*_ads_), Differential Gibbs Free Energies of Physisorption (Δ*G*), and Differential Enthalpies of Decomposition (Δ*H*_dec_) for Molecular Oxygen and Water on Pristine SnSe_2_, SnSe_1.88_, and SnSe Surfaces^a^

		physisorption		decomposition
surface	adsorbant	Δ*H*_ads_ (kJ/mol)	Δ*G* (kJ/mol)	Δ*H*_dec_ (kJ/mol)
SnSe_2_	O_2_	–17.46	–3.16	–42.28 (−161.58/∼−40.2)
	H_2_O	–13.27	18.03	220.91
SnSe_1.88_	O_2_	–37.58	–26.28	–135.67 (−99.05/–406.65)
	H_2_O	–27.93	3.37	175.61
SnSe	O_2_	–11.53	–0.23	–236.03 (−323.10/95.4)
	H_2_O	–8.12	23.18	82.22
SnO_2_ skin	H_2_O	–119.70	–106.67	–121.31

a For oxygen decomposition, the table also displays the differential enthalpy of the oxidation of the whole surface with formation of SnO and SnO_2_-like layers (in parentheses).

Considering that the yield of chemical reactions also depends on the probability of the interactions between reactants, we calculated Langmuir adsorption isotherms (Figure S12). Specifically, the combination of thermodynamic and kinetic calculations evidences that the largest part of the SnSe_*x*_ surface will be oxidized under experimental conditions (72% and 75% for SnSe_2_ and SnSe_1.88_, respectively).

On the contrary, the saturation coverage for water at room temperature is just 0.01 ML (with ML being monolayer) for SnSe_2_ and SnSe_1.88_, while the full coverage (1 ML) is reached upon exposing the SnO_2_ skin to only 5 × 10^–3^ L of H_2_O below 500 °C, thus evidencing the aptness of the SnO_2_–SnSe_2_ interface for ultrasensitive humidity sensing. The increase in temperature corresponds to a decrease in the sticking coefficient, with monolayer saturation reached at 0.05 and 10 L at 500 and 800 °C, respectively. Thus, the SnO_2_–SnSe_2_ heterostructure remains rather sensitive even at high operational temperatures.

Therefore, the SnO_2_–SnSe_2_ heterostructure shows superior chemical reactivity toward ambient species with respect to SnSe_2_. On the pristine SnSe_2_ surface, the local rearrangement of chemical bonds around each adsorbed water molecule is the origin of a redistribution of the charge density in the surface layer of SnSe_2_ with a charge transfer of 0.17 e^–^ per water molecule (Figure 2a). Correspondingly, water adsorption on SnSe_2_ and SnSe_1.88_ surfaces is energetically unfavorable for temperatures above 124 and 264 K, respectively (Figure S11). Hence, we conclude that pristine SnSe_2_ is stable in a humid environment and, consequently, is unsuitable for humidity sensing, contrarily to conclusions in refs (37), (38), and (57) On the contrary, adsorption of H_2_O on the SnO_2_–SnSe_2_ heterostructure (Figure 2b) is energetically favorable even above room temperature (Figure S11). The values of charge transferred from H_2_O to the SnO_2_ skin are 0.43 and 0.30 e^–^ for one and two H_2_O molecules per supercell, respectively. Correspondingly, DOS (Figure 2c) is modified with a direct correlation with the coverage of the adsorbate, hence proving the appropriateness for humidity sensing also at low concentrations of H_2_O.

**Figure 2:**
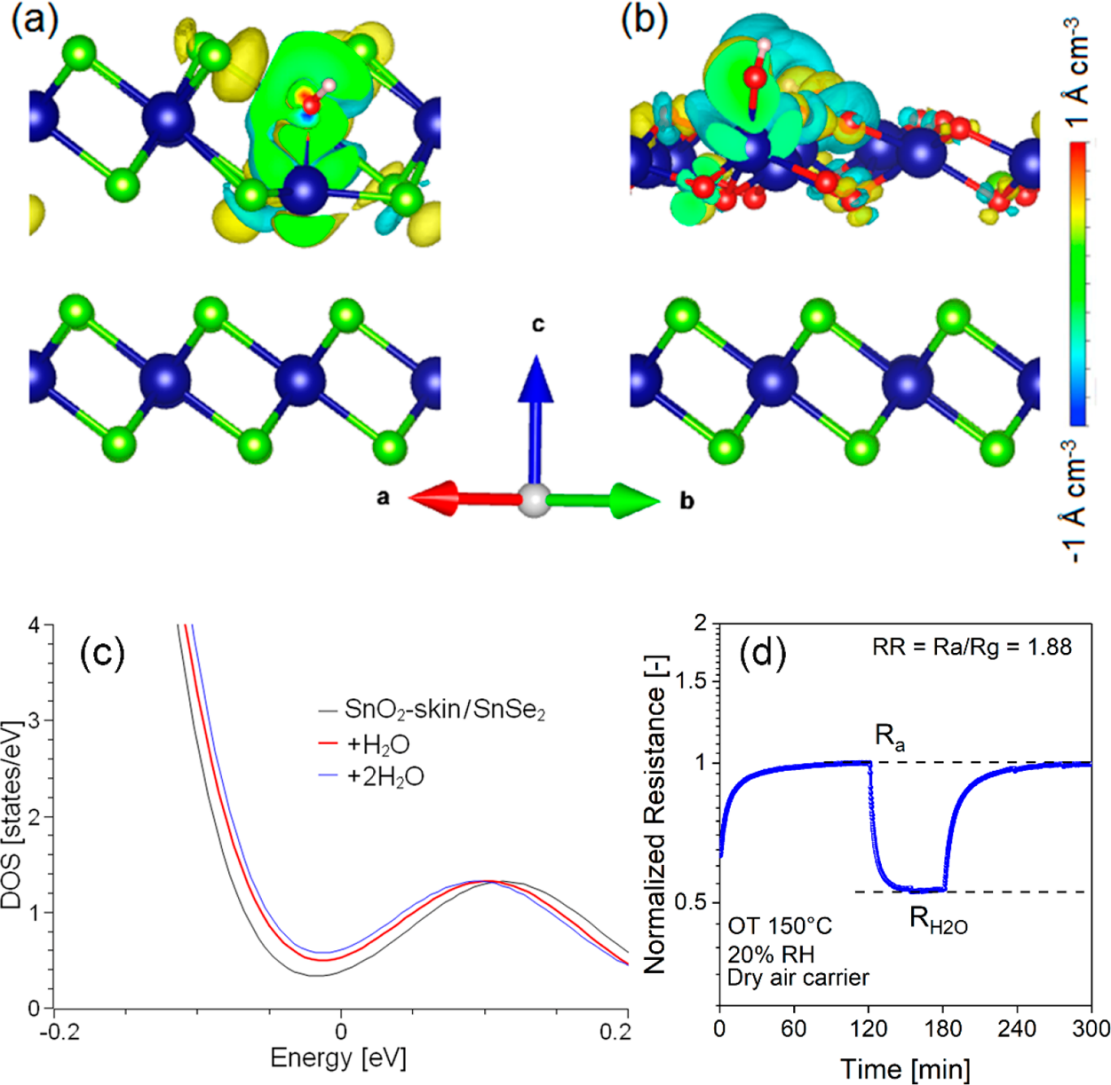
Changes in charge density after adsorption of one water molecule on (a) SnSe_2_ and (b) SnO_2_ skin-terminated SnSe_2_. Panel c represents the DOS of SnO_2_ skin-terminated SnSe_2_ (black) and of the same system modified by the adsorption of one (red) and two (blue) water molecules. The Fermi level is set at 0. Panel d shows the response of the SnSe_2_–SnO_2_ heterostructure to 20% relative humidity (RH) at an operational temperature (OT) of 150 °C (note that the average residence time of the gas in the cell is approximately 10 min).

Note that decomposition of a water molecule on the SnO_2_ skin-terminated SnSe_2_ is an exothermic process (see Table 1), although the energy gain from this process is moderate (−121.31 kJ/mol) and further water splitting is unfavorable, supporting the possible reversibility of the process.

The SnO_2_–SnSe_2_ heterostructure was tested as a humidity sensor (Figure 2d) at an operational temperature of 150 °C. Our devised humidity sensor exhibited (i) full recovery of the baseline resistance after water desorption and (ii) high sensitivity to water molecules, measured as the relative response (RR, the ratio between the resistance in dry air, *R*_a_, and *R*_H_2_O_, the resistance in a humid environment), and an experimental limit of detection (LOD) in terms of relative humidity (RH) as low as 20% (Figure 2d).

Recently, different authors^37,38,57^ have reported the outstanding performances of SnSe_2_ in humidity-sensing devices. Our findings elucidate the key role of the surface oxide skin in the interaction with a humid environment. On the contrary, in refs (37), (38), and (57), surface oxidation was not assessed; thus, the mechanism ruling humidity sensing discussed therein should be reinterpreted.

Theoretical results were validated by surface-science techniques. In particular, HREELS experiments on water-dosed Sn-based selenides (SnSe, SnSe_1.4_, SnSe_1.7_, and SnSe_2_) indicate the absence of chemisorbed water-derived species, as indicated by the lack of O–H stretching at 408–425 meV (molecular water) and 445–460 meV (hydroxyl groups) in spectra in Figure 3 (see ref (66) for more details). These findings are consistent with the positive differential Gibbs free energy of adsorption (corresponding to energetically unfavorable water adsorption) in Table 1. For the sake of comparison, we report in Figure 3 also vibrational data obtained after exposure to the same dose of H_2_O (10^5^ L, with 1 L = 1 × 10^–6^ Torr s) at room temperature the surface of other metal chalcogenides, which instead enable the stable adsorption of water molecules (PtTe_1.6_) and hydroxyl groups (InSe).

**Figure 3:**
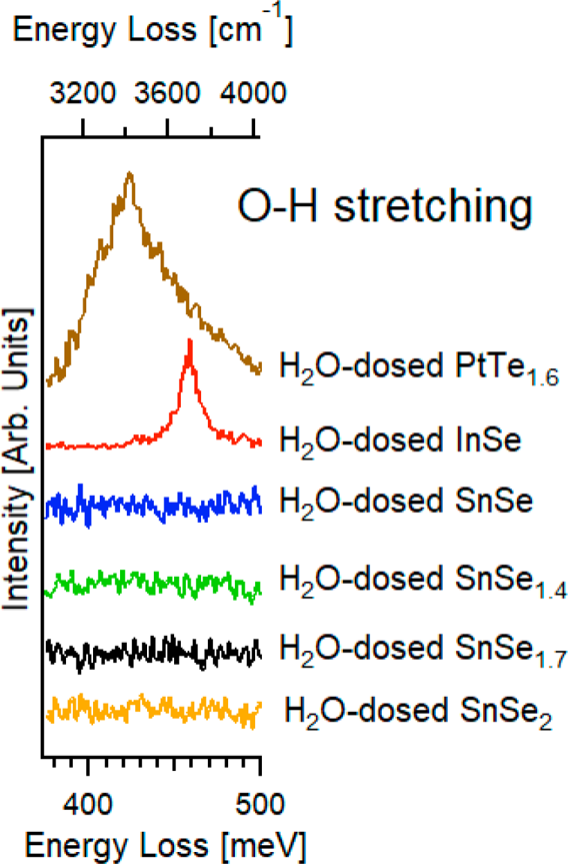
HREELS spectra in the region of the O–H band acquired after exposure to 10^5^ L of H_2_O at room temperature the surfaces of different Sn-based chalcogenides: SnSe_2_ (orange curve), SnSe_1.7_ (black), SnSe_1.4_ (green), and SnSe (blue). To provide a straightforward comparison, the figure also displays data for H_2_O-dosed InSe (red) and PtTe_1.6_ (brown) surfaces (10^5^ L at room temperature). The impinging energy is 4 eV.

The absence of reactivity toward water of Sn-based chalcogenides makes them suitable for catalysis (especially, photocatalytic water splitting^22^ and hydrogen evolution reaction^67^) and drug delivery^68^ (also considering that neither Sn nor Se is toxic). Congruently, SnSe_2_ was used as a co-catalyst in combination with TiO_2_ for hydrogen evolution reaction.^33^

Further information about the surface chemical bonds is gained by the inspection of core levels via X-ray photoelectron spectroscopy (XPS) experiments. Figure 4 shows the Sn-3d and Se-3d core levels of the SnSe_2_ single-crystal surface cleaved in ultrahigh vacuum and for the same surface modified by O_2_ and H_2_O dosage with a total dose of 10^5^ L. The Sn-3d_5/2_ core level in the as-cleaved surface displayed a binding energy (BE) of 486.8 eV (Figure 4b). Congruently, the Se-3d_5/2_ core level had a single component at a BE of 54.1 eV, in agreement with previous results for SnSe_2_^69^ and with a shift of +0.4 eV compared to the case of SnSe. Surface treatments, i.e., 10^5^ L of O_2_ and H_2_O exposure, induce only slight changes in Se-3d core levels. A novel doublet appeared in Se-3d (BE = 53.7 eV for 3d_5/2_), whose total spectral area is 5.4% (for O_2_ dosage) and 2.6% (for air exposure), arising from Se(0) segregation.^70^ In particular, from the analysis of Se-3d core-level spectra (Figure 4c), we can infer the absence of O–Se–O bonds, which would have a BE of ∼59–60 eV.^71−73^ Congruently, the intensity of the O-1s peak is especially small in SnSe_2_ exposed to both an oxidative and humid environment (Figure 4a); thus, we can evaluate the amount of oxygen to be <0.04 ML, due to a particularly weak sticking coefficient for oxygen adsorption at 300 K on SnSe_2_, with the O_2_ sticking coefficient being <10^–5^.

**Figure 4:**
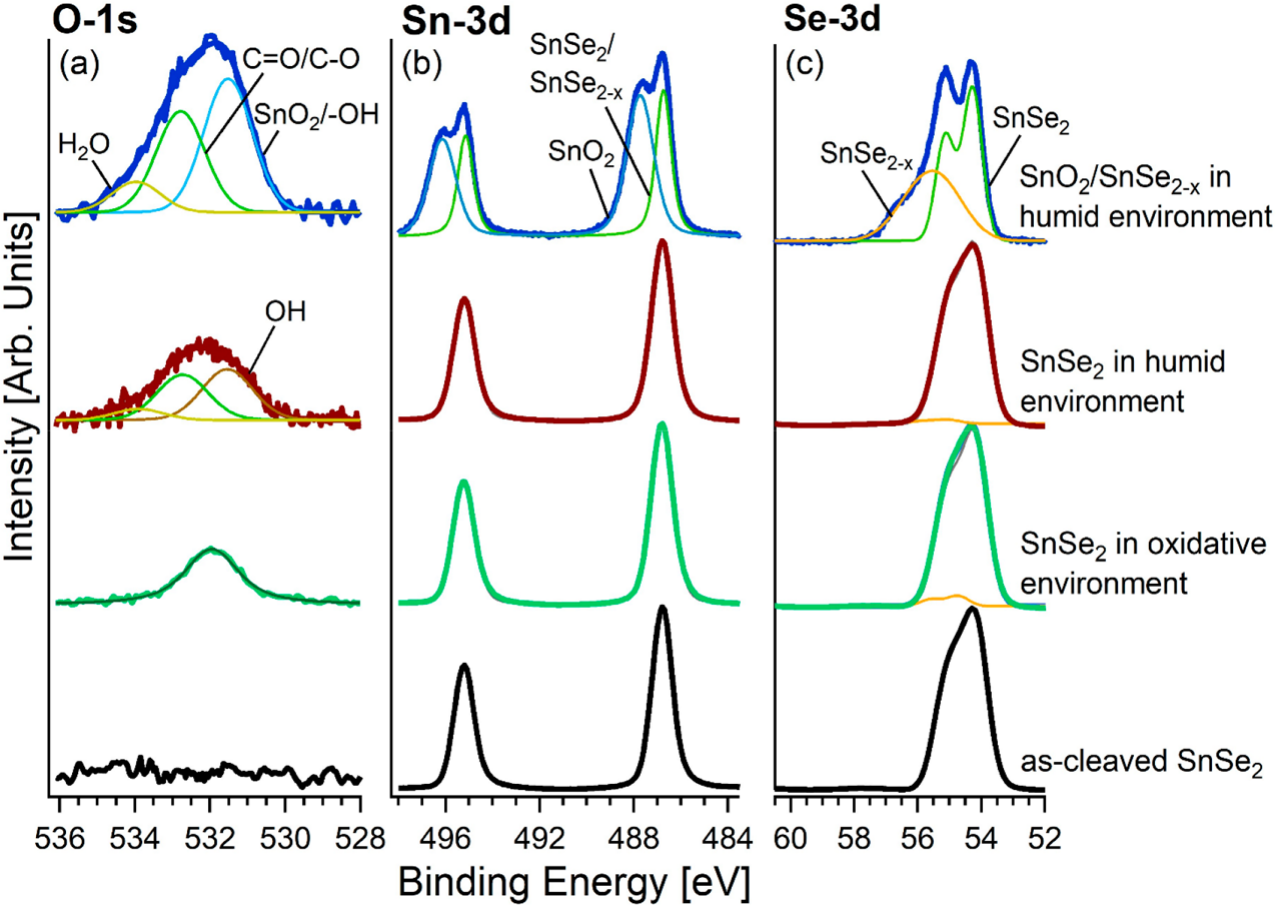
(a) O-1s, (b) Sn-3d, and (c) Se-3d core levels for the pristine surface of SnSe_2_ cleaved in situ under ultra-high-vacuum conditions and its alteration after exposure to oxidative (10^5^ L of O_2_) and humid (10^5^ L of H_2_O) environments at room temperature. The photon energy is 800 eV. We also report in each panel the corresponding spectrum for SnO_2_–SnSe_2–*x*_ exposed to a humid environment at room temperature, with *x* estimated to be 0.29.

On the contrary, we observed quite distinct peaks in SnO_2_–SnSe_2–*x*_ exposed to a humid environment (outermost spectra in the various panels of Figure 4), and a Sn-3d doublet with a *J* = ^5^/_2_ component is present at a BE of 487.8 eV, due to SnO_2_ (relative amplitude of 54%), which is consistent with previous reports for this system.^74,75^ Remarkably, no trace of O–Se–O bonds is present, as suggested by the lack of Se-3d components at 59–60 eV.^72^ This result confirms theoretical expectations that Se is involved in only a metastable oxide phase, which represents a precursor for SnO_2_ formation. Nevertheless, a broad spectral component in Se-3d suggests a different oxidation state for Se. In particular, the peak at 55.0 eV is ascribed to Se^2–^, while that one at a higher BE should be attributed to Se^–2+α^ (0 < α < 1).^76^ The analysis of survey XPS spectra enables us to evaluate α as ∼0.145, corresponding to substoichiometric SnSe_1.71_. Congruently with the results in ref (36), oxidation is feasible only in substoichiometric SnSe_2–*x*_, while perfectly stoichiometric SnSe_2_ is robust in oxidative environments, thus evidencing the pivotal role of Se vacancies in surface oxidation.

The O-1s spectrum for the SnSe_2_ surface exposed to a humid environment shows new components arising from −OH groups (relative amplitude of 45%) and H_2_O (relative amplitude of 9%) at BEs of 531.6 and 533.6 eV, respectively.^77−79^ We also exposed the SnO_2_–SnSe_2_ heterostructure to the humid environment, with the corresponding O-1s spectrum displaying the SnO_2_ component (relative amplitude of 50%) at a BE of 531.5 eV,^80,81^ overlapped with the −OH component.

In conclusion, we investigated (i) the modifications of surface properties once pristine SnSe_2_ assumes a subnanometric SnO_2_ skin upon interaction with oxidative environments and (ii) the subsequent implications for chemical sensors. Definitely, the oxidation process has a direct effect on the work function, which is increased by 0.4 eV, owing to the charge transfer between the substrate and the SnO_2_ skin of 0.56 e^–^ per SnO_2_ unit. Though the SnSe_2_ surface is inert to water at room temperature, upon surface oxidation the SnO_2_–SnSe_2_ interface shows a remarkable sensitivity to humidity. The charge transfer from H_2_O to the SnO_2_ skin is estimated to be 0.43 and 0.30 e^–^ for one and two H_2_O molecules per supercell, respectively. Correspondingly, the DOS is correlated with water coverage, hence proving the aptness for humidity sensing also at low concentrations of H_2_O. Definitely, our findings prove the significant influence of humid environments on the electrical response of the SnO_2_–SnSe_2_ heterostructure. Moreover, recent reports regarding the use of SnSe_2_ in humidity sensors should be reconsidered with regard to the physicochemical mechanism.

## Methods

Theoretical methods are described in section S8.

The single crystals were grown by the Bridgman–Stockbarger method, according to the procedure described in refs (36) and (82) (see also section S1). Their crystalline quality was secured by X-ray diffraction (XRD) (Figures S1c and S2). The analysis of the XPS survey spectrum proves the absence of contaminants in bulk crystals (Figure S3). Samples were exfoliated in situ for surface-science investigations, by using scotch tape. Gas dosage was carried out at a partial pressure of 10^–4^ mbar.

XPS experiments were carried out at the APE-HE beamline at the Elettra-Trieste synchrotron. Core-level measurements were performed with an Omicron EA125 hemispherical electron energy analyzer, with the sample at room temperature and in normal emission. Linearly polarized light formed an angle of 45° with respect to the perpendicular direction of the surface. After the subtraction of a Shirley background, Sn-3d core-level spectra were analyzed by using a Gaussian line shape convoluted with a Doniach–Sunjic function,^83^ while Se-3d and O-1s were fitted by Voigt line shapes.

HREELS experiments were performed with an Ibach-type spectrometer. The primary electron beam energy was 3.5 eV. HREELS spectra were recorded under specular conditions.

Measurements of LEEM images (Figure S4), EELS (Figure S6), and work-function changes ΔΦ (Figure 1a) were carried out at the soft X-ray beamline Nanospectroscopy at Elettra-Trieste synchrotron, using an energy-filtered LEEM–PEEM microscope with a spatial resolution of 10 nm. Specifically, measurements of ΔΦ were carried out by varying the electron beam energy across the total electron reflectivity threshold. This threshold is commonly termed the MEM–LEEM transition, which is characterized by a steep decrease in intensity as a function of a bias voltage applied to the sample (start voltage) as a decelerating potential. The ΔΦ value is identified by the shifts in the bias potential corresponding to the MEM–LEEM transition.

The gas sensing response to humidity at an operating temperature of 150 °C was determined by a volt–amperometric technique, as reported in ref (36). The RH air stream at 20% RH was obtained by mixing dry with saturated water-vapor air. In the analysis of the gas response, the relative response (RR) is defined as the ratio between the measured electrical resistance in dry air (*R*_a_) and that under 20% RH (*R*_H_2_O_).

## Supporting Information Available

Single-crystal growth (section S1), LEEM images (section S2), vibrational spectroscopy (section S3), electronic properties (section S4), density of states in pristine and defective SnSe_2_ (section S5), temperature dependence of the differential Gibbs free energy for adsorption of ambient gases (section S6), Langmuir isotherm calculations (section S7), and methods (section S8) (PDF)

## References

[ref1] Geim A. K. Nobel Lecture: Random Walk to Graphene. Rev. Mod. Phys. 2011, 83, 851–862. 10.1103/RevModPhys.83.851.

[ref2] Novoselov K. S. Nobel Lecture: Graphene: Materials in the Flatland. Rev. Mod. Phys. 2011, 83, 837–849. 10.1103/RevModPhys.83.837. 21732505

[ref3] Ambrosetti A.; Silvestrelli P. L. Trends in the Change in Graphene Conductivity Upon Gas Adsorption: The Relevance of Orbital Distortion. J. Phys. Chem. Lett. 2020, 11, 2737–2741. 10.1021/acs.jpclett.0c00379. 32202119

[ref4] Deng W.; Chen X.; Li Y.; You C.; Chu F.; Li S.; An B.; Ma Y.; Liao L.; Zhang Y. Strain Effect Enhanced Ultrasensitive MoS_2_ Nanoscroll Avalanche Photodetector. J. Phys. Chem. Lett. 2020, 11, 4490–4497. 10.1021/acs.jpclett.0c00861. 32383880

[ref5] Geng W. T.; Wang V.; Liu Y. C.; Ohno T.; Nara J. Moiré Potential, Lattice Corrugation, and Band Gap Spatial Variation in a Twist-Free MoTe_2_/MoS_2_ Heterobilayer. J. Phys. Chem. Lett. 2020, 11, 2637–2646. 10.1021/acs.jpclett.0c00605. 32188242

[ref6] Zou X.; Zhang Z.; Chen X.; Yakobson B. I. Structure and Dynamics of the Electronic Heterointerfaces in MoS_2_ by First-Principles Simulations. J. Phys. Chem. Lett. 2020, 11, 1644–1649. 10.1021/acs.jpclett.0c00147. 32046495

[ref7] Guo B. Y.; Jiang S. D.; Tang M. J.; Li K.; Sun S.; Chen P. Y.; Zhang S. Mos_2_ Membranes for Organic Solvent Nanofiltration: Stability and Structural Control. J. Phys. Chem. Lett. 2019, 10, 4609–4617. 10.1021/acs.jpclett.9b01780. 31361483

[ref8] Hu C.; Jiang Z.; Zhou W.; Guo M.; Yu T.; Luo X.; Yuan C. Wafer-Scale Sulfur Vacancy-Rich Monolayer MoS_2_ for Massive Hydrogen Production. J. Phys. Chem. Lett. 2019, 10, 4763–4768. 10.1021/acs.jpclett.9b01399. 31381350

[ref9] Yi M.; Shen Z. A Review on Mechanical Exfoliation for the Scalable Production of Graphene. J. Mater. Chem. A 2015, 3, 11700–11715. 10.1039/C5TA00252D.

[ref10] Hernandez Y.; Nicolosi V.; Lotya M.; Blighe F. M.; Sun Z.; De S.; McGovern I. T.; Holland B.; Byrne M.; Gun’ko Y. K.; Boland J. J.; Niraj P.; Duesberg G.; Krishnamurthy S.; Goodhue R.; Hutchison J.; Scardaci V.; Ferrari A. C.; Coleman J. N. High-Yield Production of Graphene by Liquid-Phase Exfoliation of Graphite. Nat. Nanotechnol. 2008, 3, 563–568. 10.1038/nnano.2008.215. 18772919

[ref11] Jin Z.; Li X.; Mullen J. T.; Kim K. W. Intrinsic Transport Properties of Electrons and Holes in Monolayer Transition-Metal Dichalcogenides. Phys. Rev. B: Condens. Matter Mater. Phys. 2014, 90, 045422 10.1103/PhysRevB.90.045422.

[ref12] Edmonds M. T.; Tadich A.; Carvalho A.; Ziletti A.; O’Donnell K. M.; Koenig S. P.; Coker D. F.; Özyilmaz B.; Neto A. H. C.; Fuhrer M. S. Creating a Stable Oxide at the Surface of Black Phosphorus. ACS Appl. Mater. Interfaces 2015, 7, 14557–14562. 10.1021/acsami.5b01297. 26126232

[ref13] Kumar A.; Telesio F.; Forti S.; Al-Temimy A.; Coletti C.; Serrano-Ruiz M.; Caporali M.; Peruzzini M.; Beltram F.; Heun S. STM Study of Exfoliated Few Layer Black Phosphorus Annealed in Ultrahigh Vacuum. 2D Mater. 2019, 6, 015005 10.1088/2053-1583/aadd20.

[ref14] Bergeron A.; Ibrahim J.; Leonelli R.; Francoeur S. Oxidation Dynamics of Ultrathin GaSe Probed through Raman Spectroscopy. Appl. Phys. Lett. 2017, 110, 241901 10.1063/1.4986189.

[ref15] Shi L.; Li Q.; Ouyang Y.; Wang J. Effect of Illumination and Se Vacancies on Fast Oxidation of Ultrathin Gallium Selenide. Nanoscale 2018, 10, 12180–12186. 10.1039/C8NR01533C. 29923588

[ref16] Fu M.; Liang L.; Zou Q.; Nguyen G. D.; Xiao K.; Li A. P.; Kang J.; Wu Z.; Gai Z. Defects in Highly Anisotropic Transition-Metal Dichalcogenide PdSe_2_. J. Phys. Chem. Lett. 2020, 11, 740–746. 10.1021/acs.jpclett.9b03312. 31880944

[ref17] Li X.; Luo N.; Chen Y.; Zou X.; Zhu H. Real-Time Observing Ultrafast Carrier and Phonon Dynamics in Colloidal Tin Chalcogenide Van Der Waals Nanosheets. J. Phys. Chem. Lett. 2019, 10, 3750–3755. 10.1021/acs.jpclett.9b01470. 31244272

[ref18] Wei Z.; Wang L.; Zhuo M.; Ni W.; Wang H.; Ma J. Layered Tin Sulfide and Selenide Anode Materials for Li- and Na-Ion Batteries. J. Mater. Chem. A 2018, 6, 12185–12214. 10.1039/C8TA02695E.

[ref19] Huang Y.; Ling C.; Liu H.; Wang S. Tuning Electronic and Magnetic Properties of SnSe_2_ Armchair Nanoribbons Via Edge Hydrogenation. J. Mater. Chem. C 2014, 2, 10175–10183. 10.1039/C4TC01919A.

[ref20] Shafique A.; Samad A.; Shin Y.-H. Ultra Low Lattice Thermal Conductivity and High Carrier Mobility of Monolayer SnS_2_ and SnSe_2_: A First Principles Study. Phys. Chem. Chem. Phys. 2017, 19, 20677–20683. 10.1039/C7CP03748A. 28737780

[ref21] Tan P.; Chen X.; Wu L.; Shang Y. Y.; Liu W.; Pan J.; Xiong X. Hierarchical Flower-Like Snse_2_ Supported Ag_3_PO_4_ Nanoparticles: Towards Visible Light Driven Photocatalyst with Enhanced Performance. Appl. Catal., B 2017, 202, 326–334. 10.1016/j.apcatb.2016.09.033.

[ref22] Fan Y.; Wang J.; Zhao M. Spontaneous Full Photocatalytic Water Splitting on 2D MoSe_2_/SnSe_2_ and WSe_2_/SnSe_2_ Vdw Heterostructures. Nanoscale 2019, 11, 14836–14843. 10.1039/C9NR03469B. 31355831

[ref23] Zeng J.; Liu E.; Fu Y.; Chen Z.; Pan C.; Wang C.; Wang M.; Wang Y.; Xu K.; Cai S.; Yan X.; Wang Y.; Liu X.; Wang P.; Liang S. J.; Cui Y.; Hwang H. Y.; Yuan H.; Miao F. Gate-Induced Interfacial Superconductivity in 1T-SnSe_2_. Nano Lett. 2018, 18, 1410–1415. 10.1021/acs.nanolett.7b05157. 29385803

[ref24] Shao Z.; Fu Z.-G.; Li S.; Cao Y.; Bian Q.; Sun H.; Zhang Z.; Gedeon H.; Zhang X.; Liu L.; Cheng Z.; Zheng F.; Zhang P.; Pan M. Strongly Compressed Few-Layered SnSe_2_ Films Grown on a SrTiO_3_ Substrate: The Coexistence of Charge Ordering and Enhanced Interfacial Superconductivity. Nano Lett. 2019, 19, 5304–5312. 10.1021/acs.nanolett.9b01766. 31287705

[ref25] Kim S.; Yao Z.; Lim J.-M.; Hersam M. C.; Wolverton C.; Dravid V. P.; He K. Lithium-Ion Batteries: Atomic-Scale Observation of Electrochemically Reversible Phase Transformations in SnSe_2_ Single Crystals. Adv. Mater. 2018, 30, 1870393 10.1002/adma.201870393. 30368925

[ref26] Bai J.; Wu H.; Wang S.; Zhang G.; Feng C.; Liu H. Synthesis of CoSe_2_-SnSe_2_ Nanocube-Coated Nitrogen-Doped Carbon (NC) as Anode for Lithium and Sodium Ion Batteries. Appl. Surf. Sci. 2019, 488, 512–521. 10.1016/j.apsusc.2019.05.096.

[ref27] Zhang F.; Xia C.; Zhu J.; Ahmed B.; Liang H.; Velusamy D. B.; Schwingenschlögl U.; Alshareef H. N. SnSe_2_ 2D Anodes for Advanced Sodium Ion Batteries. Adv. Energy Mater. 2016, 6, 1601188 10.1002/aenm.201601188.

[ref28] Zhou X.; Zhou N.; Li C.; Song H.; Zhang Q.; Hu X.; Gan L.; Li H.; Lü J.; Luo J.; Xiong J.; Zhai T. Vertical Heterostructures Based on SnSe_2_/MoS_2_ for High Performance Photodetectors. 2D Mater. 2017, 4, 025048 10.1088/2053-1583/aa6422.

[ref29] Wang M.; Wang Z.; Xu X.; Duan S.; Du C. Tin Diselenide-Based Saturable Absorbers for Eye-Safe Pulse Lasers. Nanotechnology 2019, 30, 265703 10.1088/1361-6528/ab1115. 30889561

[ref30] Zhang Y.; Liu Y.; Lim K. H.; Xing C.; Li M.; Zhang T.; Tang P.; Arbiol J.; Llorca J.; Ng K. M.; Ibáñez M.; Guardia P.; Prato M.; Cadavid D.; Cabot A. Tin Diselenide Molecular Precursor for Solution-Processable Thermoelectric Materials. Angew. Chem., Int. Ed. 2018, 57, 17063–17068. 10.1002/anie.201809847. 30398301

[ref31] Luo Y.; Zheng Y.; Luo Z.; Hao S.; Du C.; Liang Q.; Li Z.; Khor K. A.; Hippalgaonkar K.; Xu J.; Yan Q.; Wolverton C.; Kanatzidis M. G. N-Type SnSe_2_ Oriented-Nanoplate-Based Pellets for High Thermoelectric Performance. Adv. Energy Mater. 2018, 8, 1702167 10.1002/aenm.201702167.

[ref32] Sun J.; Liu S.; Wang C.; Bai Y.; Chen G.; Luo Q.; Ma F. Interface Tuning Charge Transport and Enhanced Thermoelectric Properties in Flower-Like SnSe_2_ Hierarchical Nanostructures. Appl. Surf. Sci. 2020, 510, 145478 10.1016/j.apsusc.2020.145478.

[ref33] Nasir M. S.; Yang G.; Ayub I.; Wang X.; Wang S.; Nasir A.; Yan W. Tin Diselenide Nanoflakes Decorated Hierarchical 1D TiO_2_ Fiber: A Robust and Highly Efficient Co-Catalyst for Hydrogen Evolution Reaction. Appl. Surf. Sci. 2020, 521, 146333 10.1016/j.apsusc.2020.146333.

[ref34] Lee Y. K.; Luo Z.; Cho S. P.; Kanatzidis M. G.; Chung I. Surface Oxide Removal for Polycrystalline Snse Reveals near-Single-Crystal Thermoelectric Performance. Joule 2019, 3, 719–731. 10.1016/j.joule.2019.01.001.

[ref35] Lamuta C.; Campi D.; Pagnotta L.; Dasadia A.; Cupolillo A.; Politano A. Determination of the Mechanical Properties of SnSe, a Novel Layered Semiconductor. J. Phys. Chem. Solids 2018, 116, 306–312. 10.1016/j.jpcs.2018.01.045.

[ref36] Paolucci V.; D’Olimpio G.; Kuo C.-N.; Lue C. S.; Boukhvalov D. W.; Cantalini C.; Politano A. Self-Assembled SnO_2_/SnSe_2_ Heterostructures: A Suitable Platform for Ultrasensitive NO_2_ and H_2_ Sensing. ACS Appl. Mater. Interfaces 2020, 12, 34362–34369. 10.1021/acsami.0c07901. 32662970

[ref37] Pawar M.; Kadam S.; Late D. J. High-Performance Sensing Behavior Using Electronic Ink of 2D SnSe_2_ Nanosheets. Chemistry Select 2017, 2, 4068–4075. 10.1002/slct.201700261.

[ref38] Pawbake A. S.; Date A.; Jadkar S. R.; Late D. J. Temperature Dependent Raman Spectroscopy and Sensing Behavior of Few Layer SnSe_2_ Nanosheets. Chemistry Select 2016, 1, 5380–5387. 10.1002/slct.201601347.

[ref39] Chen X.; Chen X.; Han Y.; Su C.; Zeng M.; Hu N.; Su Y.; Zhou Z.; Wei H.; Yang Z. Two-Dimensional MoSe_2_ Nanosheets Via Liquid-Phase Exfoliation for High-Performance Room Temperature NO_2_ Gas Sensors. Nanotechnology 2019, 30, 445503 10.1088/1361-6528/ab35ec. 31349238

[ref40] Guo R.; Han Y.; Su C.; Chen X.; Zeng M.; Hu N.; Su Y.; Zhou Z.; Wei H.; Yang Z. Ultrasensitive Room Temperature NO_2_ Sensors Based on Liquid Phase Exfoliated WSe_2_ Nanosheets. Sens. Actuators, B 2019, 300, 127013 10.1016/j.snb.2019.127013.

[ref41] Zhong Y.; Li W.; Zhao X.; Jiang X.; Lin S.; Zhen Z.; Chen W.; Xie D.; Zhu H. High-Response Room-Temperature NO_2_ Sensor and Ultrafast Humidity Sensor Based on SnO_2_ with Rich Oxygen Vacancy. ACS Appl. Mater. Interfaces 2019, 11, 13441–13449. 10.1021/acsami.9b01737. 30895771

[ref42] Vorokhta M.; Khalakhan I.; Vondráček M.; Tomeček D.; Vorokhta M.; Marešová E.; Nováková J.; Vlček J.; Fitl P.; Novotný M.; Hozák P.; Lančok J.; Vrňata M.; Matolínová I.; Matolín V. Investigation of Gas Sensing Mechanism of SnO_2_ Based Chemiresistor Using near Ambient Pressure Xps. Surf. Sci. 2018, 677, 284–290. 10.1016/j.susc.2018.08.003.

[ref43] Das S.; Jayaraman V. SnO_2_: A Comprehensive Review on Structures and Gas Sensors. Prog. Mater. Sci. 2014, 66, 112–255. 10.1016/j.pmatsci.2014.06.003.

[ref44] Li G.-J.; Kawi S. High-Surface-Area SnO_2_: A Novel Semiconductor-Oxide Gas Sensor. Mater. Lett. 1998, 34, 99–102. 10.1016/S0167-577X(97)00142-0.

[ref45] Di Giulio M.; Micocci G.; Serra A.; Tepore A.; Rella R.; Siciliano P. SnO_2_ Thin Films for Gas Sensor Prepared by Rf Reactive Sputtering. Sens. Actuators, B 1995, 25, 465–468. 10.1016/0925-4005(94)01397-7.

[ref46] Li W.; Kan K.; He L.; Ma L.; Zhang X.; Si J.; Ikram M.; Ullah M.; Khan M.; Shi K. Biomorphic Synthesis of 3D Mesoporous SnO_2_ with Substantially Increased Gas-Sensing Performance at Room Temperature Using a Simple One-Pot Hydrothermal Method. Appl. Surf. Sci. 2020, 512, 145657 10.1016/j.apsusc.2020.145657.

[ref47] Li W.; Ding C.; Li J.; Ren Q.; Bai G.; Xu J. Sensing Mechanism of Sb, S Doped SnO_2_(110) Surface for CO. Appl. Surf. Sci. 2020, 502, 144140 10.1016/j.apsusc.2019.144140.

[ref48] Ko W. C.; Kim K. M.; Kwon Y. J.; Choi H.; Park J. K.; Jeong Y. K. ALD-Assisted Synthesis of V_2_O_5_ Nanoislands on SnO_2_ Nanowires for Improving NO_2_ Sensing Performance. Appl. Surf. Sci. 2020, 509, 144821 10.1016/j.apsusc.2019.144821.

[ref49] Tombak A.; Ocak Y. S.; Bayansal F. Cu/SnO_2_ Gas Sensor Fabricated by Ultrasonic Spray Pyrolysis for Effective Detection of Carbon Monoxide. Appl. Surf. Sci. 2019, 493, 1075–1082. 10.1016/j.apsusc.2019.07.087.

[ref50] Han Y.; Ma Y.; Liu Y.; Xu S.; Chen X.; Zeng M.; Hu N.; Su Y.; Zhou Z.; Yang Z. Construction of MoS_2_/SnO_2_ Heterostructures for Sensitive NO_2_ Detection at Room Temperature. Appl. Surf. Sci. 2019, 493, 613–619. 10.1016/j.apsusc.2019.07.052.

[ref51] Barsan N.; Weimar U. Understanding the Fundamental Principles of Metal Oxide Based Gas Sensors; the Example of CO Sensing with SnO_2_ Sensors in the Presence of Humidity. J. Phys.: Condens. Matter 2003, 15, R813 10.1088/0953-8984/15/20/201.

[ref52] Choi K.-I.; Hübner M.; Haensch A.; Kim H.-J.; Weimar U.; Barsan N.; Lee J.-H. Ambivalent Effect of Ni Loading on Gas Sensing Performance in SnO_2_ Based Gas Sensor. Sens. Actuators, B 2013, 183, 401–410. 10.1016/j.snb.2013.04.007.

[ref53] Shelke N. T.; Late D. J. Hydrothermal Growth of MoSe_2_ Nanoflowers for Photo- and Humidity Sensor Applications. Sens. Actuators, A 2019, 295, 160–168. 10.1016/j.sna.2019.05.045.

[ref54] Gupta S. P.; Pawbake A. S.; Sathe B. R.; Late D. J.; Walke P. S. Superior Humidity Sensor and Photodetector of Mesoporous ZnO Nanosheets at Room Temperature. Sens. Actuators, B 2019, 293, 83–92. 10.1016/j.snb.2019.04.086.

[ref55] Theillet P.-O.; Pierron O. Quantifying Adsorbed Water Monolayers on Silicon Mems Resonators Exposed to Humid Environments. Sens. Actuators, A 2011, 171, 375–380. 10.1016/j.sna.2011.09.002.

[ref56] Panchal V.; Giusca C. E.; Lartsev A.; Martin N. A.; Cassidy N.; Myers-Ward R. L.; Gaskill D. K.; Kazakova O. Atmospheric Doping Effects in Epitaxial Graphene: Correlation of Local and Global Electrical Studies. 2D Mater. 2016, 3, 015006 10.1088/2053-1583/3/1/015006.

[ref57] Tannarana M.; Pataniya P. M.; Bhakhar S. A.; Solanki G. K.; Valand J.; Narayan S.; Patel K. D.; Jha P. K.; Pathak V. M. Humidity Sensor Based on Two-Dimensional SnSe_2_/MWCNTs Nanohybrid for the Online Monitoring of Human Respiration and Touchless Positioning Interface. ACS Sustainable Chem. Eng. 2020, 8, 12595–12602. 10.1021/acssuschemeng.0c04027.

[ref58] Nataf G. F.; Grysan P.; Guennou M.; Kreisel J.; Martinotti D.; Rountree C. L.; Mathieu C.; Barrett N. Low Energy Electron Imaging of Domains and Domain Walls in Magnesium-Doped Lithium Niobate. Sci. Rep. 2016, 6, 33098 10.1038/srep33098. 27608605 PMC5016809

[ref59] Leung T.; Kao C.; Su W.; Feng Y.; Chan C. Relationship between Surface Dipole, Work Function and Charge Transfer: Some Exceptions to an Established Rule. Phys. Rev. B: Condens. Matter Mater. Phys. 2003, 68, 195408 10.1103/PhysRevB.68.195408.

[ref60] Roy T.; Tosun M.; Hettick M.; Ahn G. H.; Hu C.; Javey A. 2D-2D Tunneling Field-Effect Transistors Using WSe_2_/SnSe_2_ Heterostructures. Appl. Phys. Lett. 2016, 108, 083111 10.1063/1.4942647.

[ref61] Li F.; Gao X.; Wang R.; Zhang T.; Lu G. Study on TiO_2_-SnO_2_ Core-Shell Heterostructure Nanofibers with Different Work Function and Its Application in Gas Sensor. Sens. Actuators, B 2017, 248, 812–819. 10.1016/j.snb.2016.12.009.

[ref62] Batzill M.; Katsiev K.; Burst J. M.; Losovyj Y.; Bergermayer W.; Tanaka I.; Diebold U. Tuning Surface Properties of SnO_2_(101) by Reduction. J. Phys. Chem. Solids 2006, 67, 1923–1929. 10.1016/j.jpcs.2006.05.042.

[ref63] Serna M. I.; Hasan S. M.; Nam S.; El Bouanani L.; Moreno S.; Choi H.; Alshareef H. N.; Minary-Jolandan M.; Quevedo-Lopez M. A. Low-Temperature Deposition of Layered SnSe_2_ for Heterojunction Diodes. Adv. Mater. Interfaces 2018, 5, 1800128 10.1002/admi.201800128.

[ref64] Zhang Q.; Li M.; Lochocki E. B.; Vishwanath S.; Liu X.; Yan R.; Lien H.-H.; Dobrowolska M.; Furdyna J.; Shen K. M.; et al. Band Offset and Electron Affinity of Mbe-Grown SnSe_2_. Appl. Phys. Lett. 2018, 112, 042108 10.1063/1.5016183.

[ref65] Bauer E. Surface Microscopy with Low Energy Electrons; Springer, 2014; Vol. 23.

[ref66] Henderson M. A. The Interaction of Water with Solid Surfaces: Fundamental Aspects Revisited. Surf. Sci. Rep. 2002, 46, 1–308. 10.1016/S0167-5729(01)00020-6.

[ref67] Inamdar A. N.; Som N. N.; Pratap A.; Jha P. K. Hydrogen Evolution and Oxygen Evolution Reactions of Pristine and Alkali Metal Doped Snse_2_ Monolayer. Int. J. Hydrogen Energy 2020, 45, 18657–18665. 10.1016/j.ijhydene.2019.07.093.

[ref68] Deng J.; Mo Y.; Liu J.; Guo R.; Zhang Y.; Xue W.; Zhang Y. In Vitro Study of SnS_2_, BiOCl and SnS_2_-Incorporated BiOCl Inorganic Nanoparticles Used as Doxorubicin Carrier. J. Nanosci. Nanotechnol. 2016, 16, 5740–5745. 10.1166/jnn.2016.11745. 27427625

[ref69] Wu S.; Liu C.; Wu Z.; Miao L.; Gao J.; Hu X.; Chen J.; Zheng Y.; Wang X.; Shen C.; et al. Realizing Tremendous Electrical Transport Properties of Polycrystalline SnSe_2_ by Cl-Doped and Anisotropy. Ceram. Int. 2019, 45, 82–89. 10.1016/j.ceramint.2018.09.136.

[ref70] Nagaraju G.; Cha S. M.; Sekhar S. C.; Yu J. S. Metallic Layered Polyester Fabric Enabled Nickel Selenide Nanostructures as Highly Conductive and Binderless Electrode with Superior Energy Storage Performance. Adv. Energy Mater. 2017, 7, 1601362 10.1002/aenm.201601362.

[ref71] Dimitriev Y.; Yordanov St.; Lakov L. The Structure of Oxide Glasses Containing SeO_2_. J. Non-Cryst. Solids 2001, 293–295, 410–415. 10.1016/S0022-3093(01)00836-5.

[ref72] Bachvarova-Nedelcheva A.; Iordanova R.; Kostov K. L.; Yordanov S.; Ganev V. Structure and Properties of a Non-Traditional Glass Containing TeO_2_, SeO_2_ and MoO_3_. Opt. Mater. 2012, 34, 1781–1787. 10.1016/j.optmat.2012.05.002.

[ref73] Fan Y.; Zhuo Y.; Li L. Seo_2_ Adsorption on Cao Surface: Dft and Experimental Study on the Adsorption of Multiple SeO_2_ Molecules. Appl. Surf. Sci. 2017, 420, 465–471. 10.1016/j.apsusc.2017.04.233.

[ref74] Al-Hada N. M.; Kamari H. M.; Baqer A. A.; Shaari A. H.; Saion E. Thermal Calcination-Based Production of SnO_2_ Nanopowder: An Analysis of Sno_2_ Nanoparticle Characteristics and Antibacterial Activities. Nanomaterials 2018, 8, 250 10.3390/nano8040250. PMC5923580 29673195

[ref75] Zhang W.; Li M.; Xiao X.; Huang X.; Jiang Y.; Fan X.; Chen L. In Situ Synthesis of Ultrasmall Sno_2_ Quantum Dots on Nitrogen-Doped Reduced Graphene Oxide Composite as High Performance Anode Material for Lithium-Ion Batteries. J. Alloys Compd. 2017, 727, 1–7. 10.1016/j.jallcom.2017.04.316.

[ref76] Wakita T.; Paris E.; Kobayashi K.; Terashima K.; Hacisalihoǧlu M. Y.; Ueno T.; Bondino F.; Magnano E.; Píš I.; Olivi L.; Akimitsu J.; Muraoka Y.; Yokoya T.; Saini N. L. The Electronic Structure of Ag_1-X_Sn_1+X_Se_2_ (X = 0.0, 0.1, 0.2, 0.25 and 1.0). Phys. Chem. Chem. Phys. 2017, 19, 26672–26678. 10.1039/C7CP05369J. 28967026

[ref77] Hoch L. B.; Wood T. E.; O’Brien P. G.; Liao K.; Reyes L. M.; Mims C. A.; Ozin G. A. The Rational Design of a Single-Component Photocatalyst for Gas-Phase Co_2_ Reduction Using Both Uv and Visible Light. Adv. Sci. 2014, 1, 1400013 10.1002/advs.201400013. PMC5115262 27980897

[ref78] Detweiler Z. M.; Wulfsberg S. M.; Frith M. G.; Bocarsly A. B.; Bernasek S. L. The Oxidation and Surface Speciation of Indium and Indium Oxides Exposed to Atmospheric Oxidants. Surf. Sci. 2016, 648, 188–195. 10.1016/j.susc.2015.10.026.

[ref79] Nappini S.; Matruglio A.; Naumenko D.; Dal Zilio S.; Bondino F.; Lazzarino M.; Magnano E. Graphene Nanobubbles on TiO_2_ for *in-Operando* Electron Spectroscopy of Liquid-Phase Chemistry. Nanoscale 2017, 9, 4456–4466. 10.1039/C6NR09061C. 28304018

[ref80] Hong X.; Li S.; Wang R.; Fu J. Hierarchical SnO_2_ Nanoclusters Wrapped Functionalized Carbonized Cotton Cloth for Symmetrical Supercapacitor. J. Alloys Compd. 2019, 775, 15–21. 10.1016/j.jallcom.2018.10.099.

[ref81] Xu H.; Ju J.; Li W.; Zhang J.; Wang J.; Cao B. Superior Triethylamine-Sensing Properties Based on TiO_2_/SnO_2_ N–N Heterojunction Nanosheets Directly Grown on Ceramic Tubes. Sens. Actuators, B 2016, 228, 634–642. 10.1016/j.snb.2016.01.059.

[ref82] Guo C.; Guo W.; Xu H.; Zhang L.; Chen G.; D'Olimpio G.; Kuo C.-N.; Lue C. S.; Wang L.; Politano A.; Chen X.; Lu W. Ultrasensitive Ambient-Stable SnSe_2_-Based Broadband Photodetectors for Room-Temperature IR/THz Energy Conversion and Imaging. 2D Mater. 2020, 7, 035026 10.1088/2053-1583/ab8ec0.

[ref83] Doniach S.; Sunjic M. Many-Electron Singularity in X-Ray Photoemission and X-Ray Line Spectra from Metals. J. Phys. C: Solid State Phys. 1970, 3, 285 10.1088/0022-3719/3/2/010.

