# Supplementary material for: Charge Redistribution Mechanisms in SnSe2 Surfaces Exposed to Oxidative and Humid Environments and Their Related Influence on Chemical Sensing
Source: J Phys Chem Lett. 2020 Oct 9;11(21):9003–11. doi: 10.1021/acs.jpclett.0c02616 (PMC8015219; doi:10.1021/acs.jpclett.0c02616)
Supplement: Supplementary file 1 — jz0c02616_si_001.pdf [file jz0c02616_si_001.pdf]

SUPPORTING INFORMATION

Charge Redistribution Mechanisms in $SnSe_2$ Surfaces Exposed to Oxidative and Humid Environments and their Related Influence on Chemical Sensing

Gianluca D'Olimpio[1], Francesca Genuzio[2], Tevfik Onur Menteş[2], Valentina Paolucci[3], Chia-Nung Kuo[4,], Amjad Al Taleb[5], Chin Shan Lue[4], Piero Torelli[2,6], Daniel Farías[5,7,8], Andrea Locatelli[2], Danil W. Boukhvalov[9,10], Carlo Cantalini[3,], and Antonio Politano[1,11,]**

[1] Department of Physical and Chemical Sciences, University of L'Aquila, via Vetoio, 67100 L'Aquila (AQ), Italy
[2] Elettra-Sincrotrone S.C.p.A, S.S. 14-km 163.5 in AREA Science Park 34149, Trieste, Italy
[3] Department of Industrial and Information Engineering and Economics, Via G. Gronchi 18, University of L'Aquila, I-67100 L'Aquila, Italy
[4] Department of Physics, National Cheng Kung University, 1 Ta-Hsueh Road, 70101 Tainan, Taiwan
[5] Departamento de Física de la Materia Condensada, Universidad Autónoma de Madrid, 28049 Madrid, Spain
[6] Consiglio Nazionale delle Ricerche (CNR)- Istituto Officina dei Materiali (IOM), Laboratorio TASC in Area Science Park S.S. 14 km 163.5 34149 Trieste, Italy
[7] Instituto 'Nicolás Cabrera', Universidad Autónoma de Madrid, 28049 Madrid, Spain
[8] Condensed Matter Physics Center (IFIMAC), Universidad Autónoma de Madrid, 28049 Madrid, Spain
[9] College of Science, Institute of Materials Physics and Chemistry, Nanjing Forestry University, Nanjing 210037, P. R. China
[10] Theoretical Physics and Applied Mathematics Department, Ural Federal University, Mira Street 19, 620002 Ekaterinburg, Russia
[11] CNR-IMM Istituto per la Microelettronica e Microsistemi, VIII strada 5, I-95121 Catania, Italy

S1. Characterization of grown single crystals

Centimeter-scale single crystals displaying flat surfaces (Fig. S1c) were grown by Bridgman method. A mixture of Sn (4N pieces) and Se (5N pieces) elements with a stoichiometric ratio of 1:2 was loaded into an evacuated quartz ampoule. The growth was conducted in a vertical two-zone tube furnace with a thermal gradient of 10 K/cm. The charged ampoule was heated up to 973 K in 12 h, kept at 973 K for 24 h and then slowly cooled to 903 K with a rate of 1 K/h. The resulting ingot with typical dimension of several mm^2 were cleaved easily along the basal plane. The phase purity and the crystal structure of obtained crystals were characterized by powder X-ray diffraction

(XRD), with the result given in Fig. S1c. All peaks in the diffraction pattern can be indexed with the CdI_2–type structure (space group P-3m1). The determined lattice parameters a = 0.3804 and c = 0.6128 nm are consistent with previous reports[1-2]. We also carried out the XRD and Laue diffraction measurements on single crystals. Samples were exfoliated in situ by adhesive tape.

The Raman spectrum of the grown $SnSe_2$ single crystal (Fig. S1d) shows the E_g and A_{1g} modes at 109 and 184 cm^{-1}, respectively, congruently with previous reports [3-4]. The narrow (00l) diffraction peaks (Fig. S1c), as well as the sharp spots in the Laue pattern (inset of Fig. S2) reveal the excellent crystallinity for our $SnSe_2$ crystals. The XRD pattern of powderized single crystal is shown in Fig. S2.

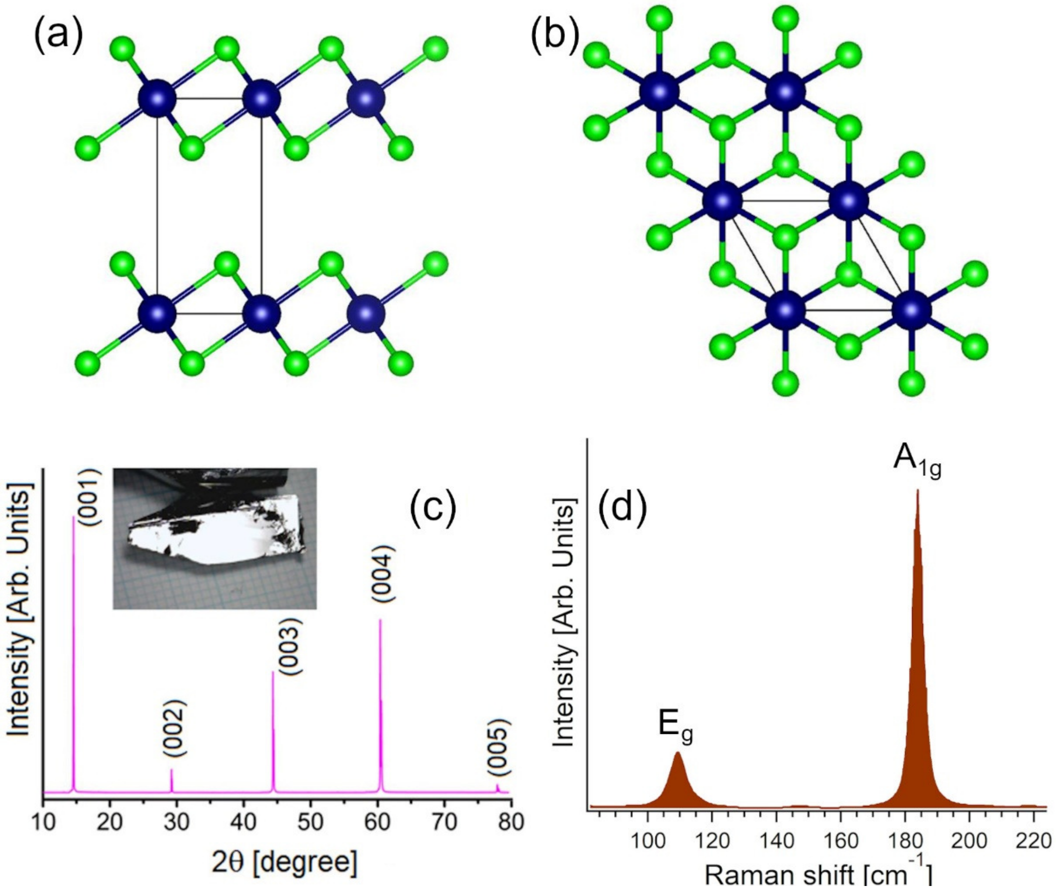

Figure S1: (a) Side and (b) top views of the atomic structure of $SnSe_2$. Green and blue balls denote Se and Sn atoms, respectively. Panel (c) reports the single-crystal XRD pattern from the (001) plane of $SnSe_2$. The inset shows a photograph of an as-grown $SnSe_2$ single crystal. Panel (d) reports the Raman spectrum of $SnSe_2$ single crystal acquired at room temperature with a laser with wavelength λ= 632.8 nm.

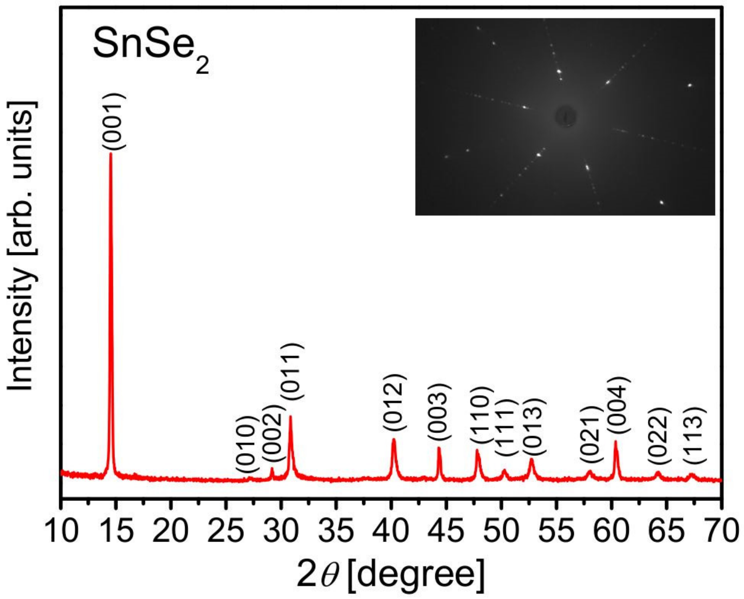

Figure S2: XRD pattern of powder $SnSe_2$, taken with Cu K_α. The inset shows the Laue diffraction image taken along the (001) direction.

The survey XPS spectrum shows that the single crystal has no contaminants in the bulk (Fig. S3).

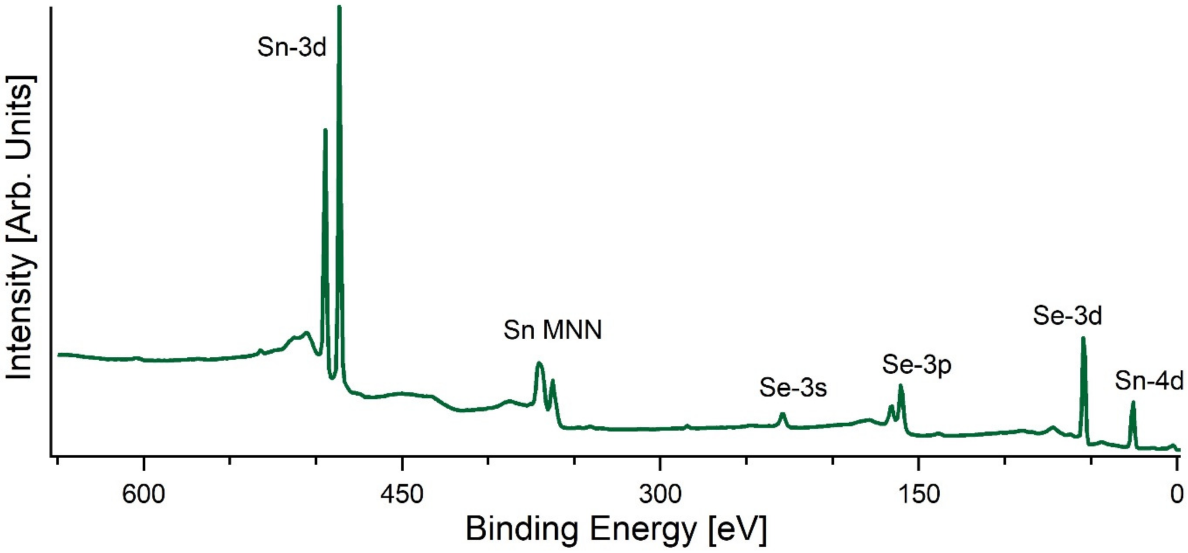

Figure S3: Survey XPS spectrum of $SnSe_2$ single crystal with a photon energy of 800 eV, acquired at APE-HE beamline Elettra synchrotron.

S2. LEEM image

Figure S4 reports a LEEM image of the as-cleaved $SnSe_2$ surface. Note that the surface displays terraces in the micrometer range.

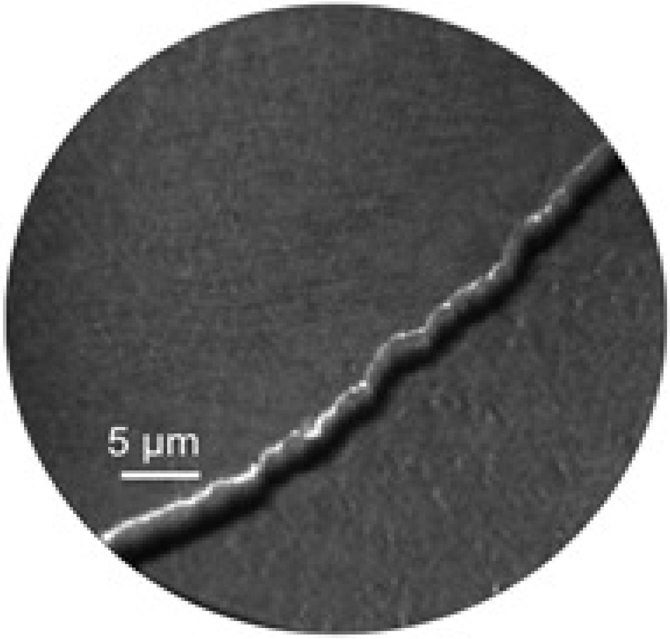

Figure S4: LEEM image of the $SnSe_2$ sample (kinetic energy of 1 eV).

We also studied the formation of the surface oxide layer. Figure S5 shows (a) the as-cleaved surface and its modification after an exposure to (b) 700 L of O_2 in vacuum and (c) to air. The comparison of the three images reveals that while the oxidation in vacuum does not affects significantly the surface (Fig. S5c), air exposure induces changes in arising from the modification of the work function energy (see Fig. 1a and its related discussion).

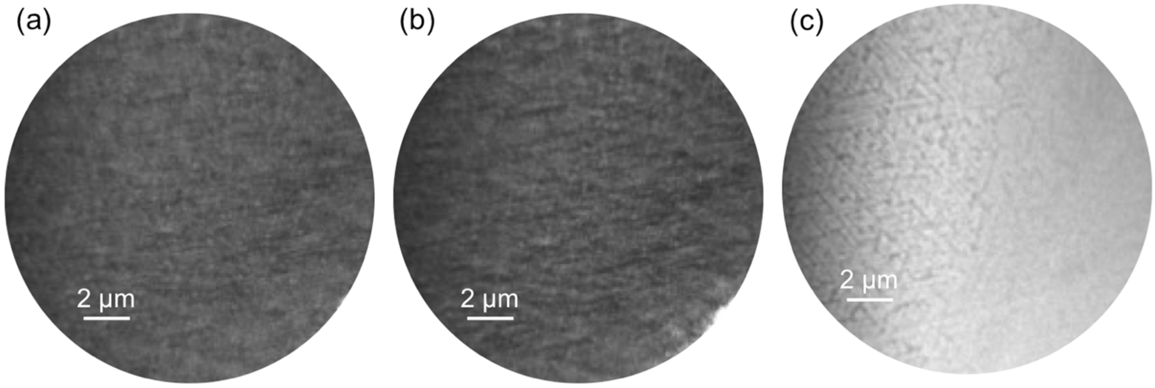

Figure S5: LEEM images for (a) as-cleaved, (b) O_2-dosed; and (c) air-exposed $SnSe_2$ surfaces. The intensity gradient in (c) arises from the local $\Delta\Phi$.

S3. Vibrational spectroscopy

Further evidence of the presence of SnO_2 is provided by vibrational spectroscopy. Remarkably, the vibrational spectrum of the oxidized $SnSe_{1.7}$(001) surface (Fig. S6) nearly coincides with the phonon spectrum of SnO_2(110), with surface optical phonons and their combination mode[5]. Precisely, modes

at 47, 98, 125, 176 and 219 meV were observed. It should be noted that the features at 47 and 98 meV are blue-shifted by 5 and 11 meV in the disordered tin-oxide layer formed in the oxidation of $SnSe_2$ with respect to their value for bulk crystalline $SnO_2(110)$ [5]. These modes correspond to the A_{2g} and the B_{2g} phonons, respectively, in agreement with calculations in Ref. [6].
The formed SnO_2 skin is amorphous, as indicated by the disappearance of diffraction peaks in the microprobe low-energy electron diffraction pattern.

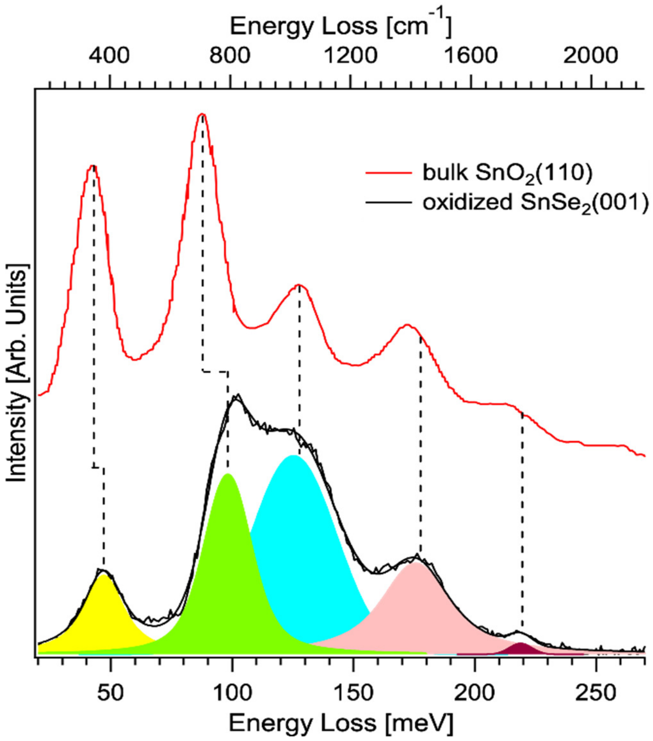

Figure S6: Vibrational spectra for the oxidized surface of $SnSe_2(001)$. Primary electron beam energy is 4 eV. Experiments were carried out at room temperature. For the sake of comparison, we report also data for bulk $SnO_2(110)$ (data taken from Ref. [5]).

S4. Electronic properties

Further information on physicochemical properties of the $SnO_2/SnSe_2$ heterostructure could be achieved by analyzing the excitation spectrum. Especially, electron energy loss spectroscopy (EELS), extended up to the ultraviolet range of the electromagnetic spectrum (Fig. S7), enables monitoring the surface status with a technique with probing depth as low as (0.9±0.1) nm in our experimental conditions [7], which is lower by more than 10^2 with respect to optical techniques. Specifically, the excitation spectrum of the as-cleaved $SnSe_2$ surface shows a main feature at 15.9 eV with a shoulder at 12.0 eV, ascribed to interband transitions from Se-4s core levels and,

moreover, two weak losses at 7.5 and 26.8 eV. The excitation spectrum of the air-exposed $SnSe_2$ sample is dominated by an emerging broad mode centered around ~18 eV, with two weak peaks at 7.5 and 26.8 eV, evidently insensitive to surface modification. Notably, polycrystalline SnO_2 films display the feature at 18.0 eV. Precisely, this feature was previously attributed to sub-oxide SnO_{2-x} phases [8]. However, the inspection of density of states (DOS) in Fig. S8 reveals that the mode at 18.0 eV is related to a single-particle transition starting from O-2s band in SnO_2. Definitely, oxidation induces the appearance of O-2s contributions around -18 eV and of states in the nearness of the Fermi level (Fig. S8), with a subsequent decrease of the magnitude of the differential enthalpy of oxidation (see Tab. 1).

For the sake of completeness, we mention that the weak peaks at 7.5 and 26.8 eV are ascribed to interband transitions originated by Sn-5s and Se-3s levels, respectively.

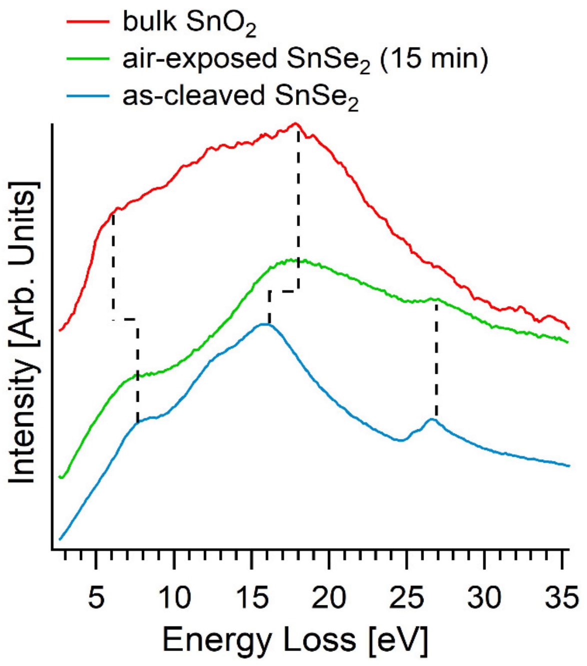

Figure S7: Excitation spectrum, probed for a primary electron beam energy of 300 eV, for as-cleaved $SnSe_2$ and the same surface exposed to air for 15 minutes. For the sake of comparison, we report also data for bulk SnO_2 (data taken from Ref. [5]).

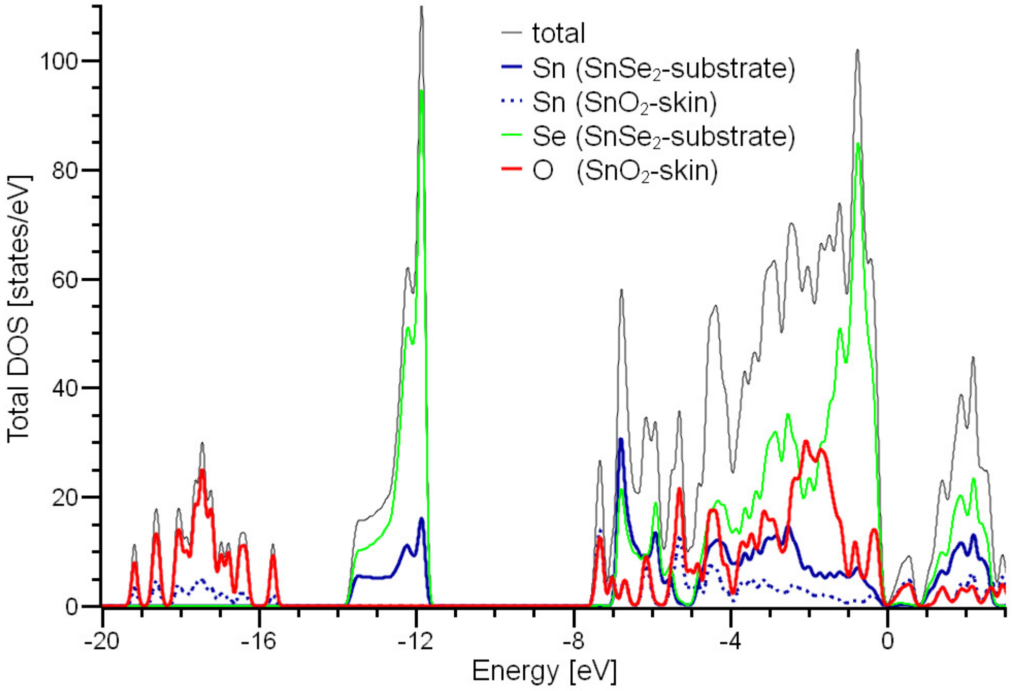

Figure S8: Partial densities of states for $SnSe_2$ slab with SnO_2-skin (see Fig. 2d). Fermi energy is set as zero.

S5. Density of states in pristine and defective $SnSe_2$

Calculations demonstrate that the presence of the Se vacancies provides insignificant changes in the electronic structure of $SnSe_2$ (Fig. S9a). On the other hand, the oxygenation of the surface, without formation of SnO_2-skin, induces the appearance of states around the Fermi level without other visible changes in the electronic structure. Only the formation of the SnO_2 skin leads to a decrease of Se *3p* peak at -1 eV and the flattening of the valence band, due to contributions from O-*2p* states (Fig. S9b). Obtained results are in qualitative agreement with experimental observation of broadening of feature in valence band, with a decrease of the density of states around the Fermi level (Fig. S10).

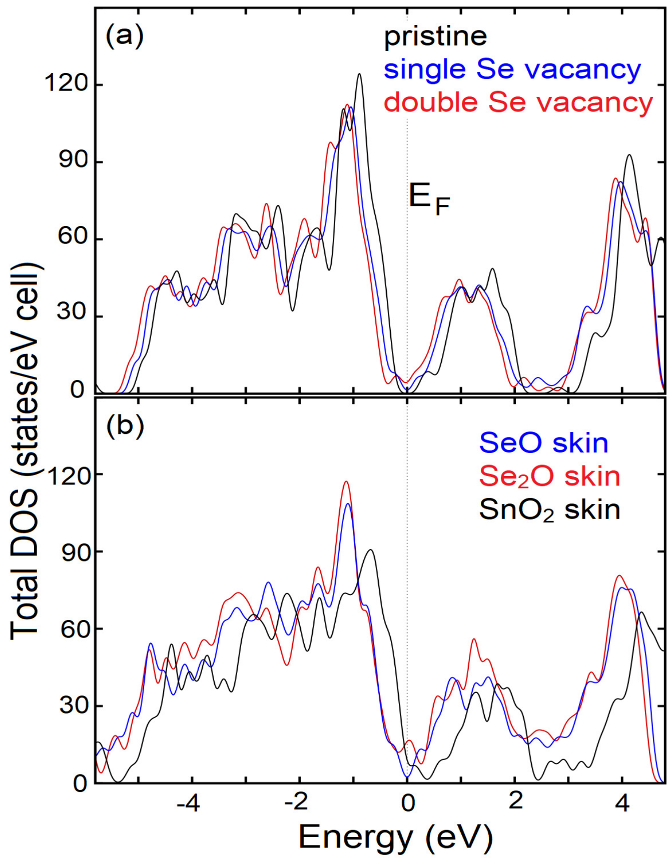

Figure S9: Densities of perfect and defected $SnSe_2$ surface (a) and perfect surface after formation of various oxides skins (see Fig. 2a,b).

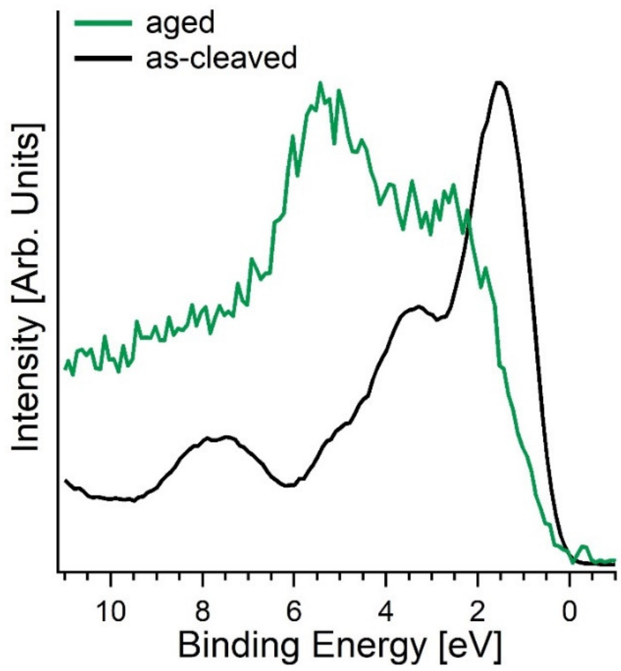

Figure S10: Valence-band spectra for as-cleaved and aged (one week) single crystal of $SnSe_2$.

S6. Temperature dependence of the differential Gibbs free energy for adsorption of ambient gases

Figure S11 represents the behavior of the differential Gibbs free energy for adsorption of ambient gases (oxygen and water) on $SnSe_2$, SnSe1.$_{88}$ and SnO_2-skin as a function of temperature. Notably, water adsorption on SnO_2-skin is energetically favorable even at high temperature. On the contrary, for $SnSe_2$ and SnSe1.$_{88}$

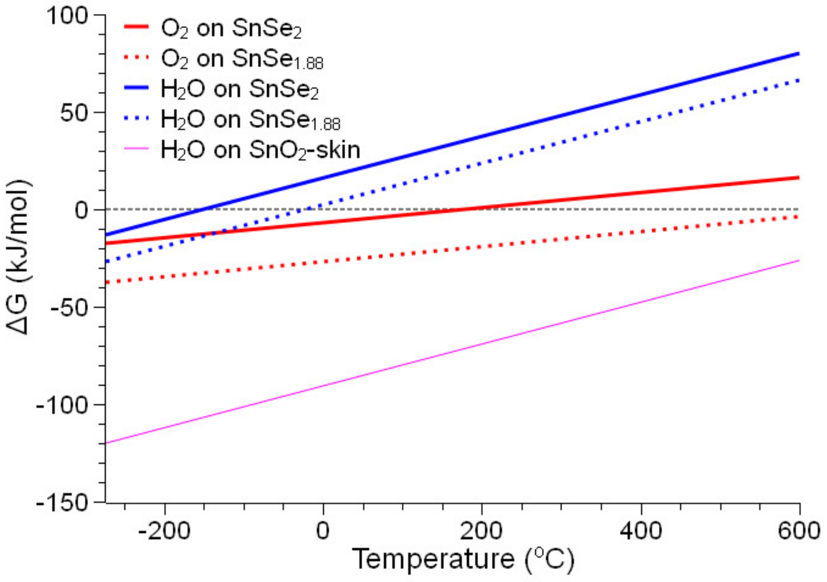

Figure S11: Behavior of the differential Gibbs free energy of physical adsorption of molecular oxygen and water on $SnSe_2$, SnSe1.$_{88}$ and SnO_2-skin as function of temperature.

S7. Langmuir isotherms calculations

Considering that the yield of chemical reactions also depends on the probability of the interactions between reactants, we calculated Langmuir adsorption isotherms.

To check the surface coverage obtained by exposure we calculate Langmuir isotherms by standard formulas:

$$q = \sum_i q_i \frac{K_i P}{1+K_i P},$$

where q_i is the part of active sites of selected type, P pressure and K_i is equilibrium constants for adsorption and desorption at given temperature calculated by Van't Hoff equation:

$$K_i = e^{(-\frac{\Delta G_i}{RT})} - e^{(\frac{\Delta G_i}{RT})},$$

where ΔG_i is the free energy of adsorption.

Specifically, the combination of thermodynamic and kinetic calculations evidence that the largest part of $SnSe_x$ surface will be oxidized at experimental conditions (72% and 75% for $SnSe_2$ and $SnSe_{1.88}$, respectively).

On the other hand, the saturation coverage for water at room temperature is just 0.01 ML (with ML being monolayer) for $SnSe_2$ and $SnSe_{1.88}$, while the full coverage (1 ML) is reached upon exposing the SnO_2 skin to only $5 \cdot 10^{-3}$ L of H_2O for temperature below 500°C, thus evidencing the aptness of $SnO_2/SnSe_2$ for ultrasensitive humidity sensing. The increase of the temperature corresponds to a decrease of the sticking coefficient, with monolayer saturation reached at 0.05 and 10 L at 500 and 800 °C, respectively. Thus, the $SnO_2/SnSe_2$ heterostructure remains rather sensitive even at high operational temperatures.

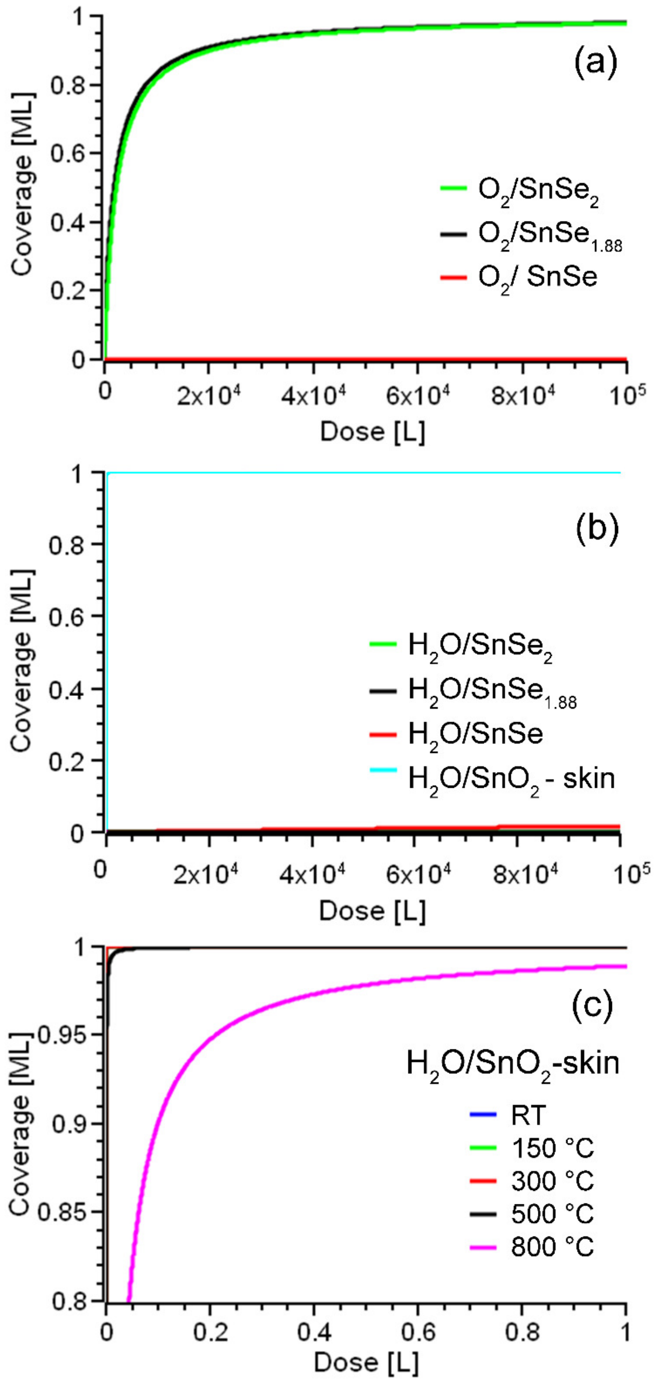

Figure S12: Langmuir isotherms for (a) O_2 adsorption at room temperature; (b) H_2O adsorption at room temperature; (c) H_2O/SnO_2-skin at various temperatures.

S8. Methods

Experimental methods

XPS experiments were performed at the High-Energy branch of the Advanced Photoelectric Experiments beamline (APE-HE) of the Elettra Synchrotron, Trieste, Italy. XPS spectra were acquired with an Omicron EA125 hemispherical electron energy analyzer, with the sample at room temperature and in normal emission condition. The linearly polarized light was impinging on the sample forming an angle of 45 degrees with respect to the normal to the surface. Under our experimental conditions, we had any evidence of beam-induced damage even after long-time exposure to synchrotron light.

HREELS experiments were carried out with a Delta 0.5 spectrometer by Specs GmbH, Germany. The impinging energy is 3.5 eV. Spectra were recorded in specular direction, with an incidence angle of 55° with respect to the sample normal.

LEEM images, EELS and $\Delta\Phi$ measurements were carried out at the soft X-ray beamline Nanospectroscopy at Elettra Synchrotron, Trieste, Italy, using an energy filtered LEEM-PEEM microscopy. The SPELEEM III microscope (Elmitec GmbH) combines laterally resolved XPS with LEEM. In LEEM mode, the microscope probes the specimen with low-energy electrons, using the elastically backscattered electron beam for imaging. Such a LEEM operation allows surface-sensitive real-space imaging with a best spatial resolution of about 10 nm. EELS measurements were acquired at normal incidence, by varying the bias voltage of the electron energy filter, so that it is possible to scan the kinetic energy of the inelastically reflected beam reaching the detector, obtaining the EELS spectrum.

Measurements of $\Delta\Phi$ were carried out by varying the electron beam energy across the total electron reflectivity threshold. This threshold is commonly termed as MEM-LEEM transition, which is characterized by a steep drop of intensity as a function of a bias voltage applied to the sample (start voltage) as a decelerating potential. The $\Delta\Phi$ value is identified by the shifts in the bias potential corresponding to the MEM-LEEM transition.

Theoretical methods

The atomic structure and energetics of various configurations of various gases on $SnSe_2$ were studied by DFT using the QUANTUM-ESPRESSO code [9] and the GGA–PBE + van der Waals (vdW) approximation, feasible for the description of the adsorption of molecules on surfaces [10-11]. We used energy cutoffs of 25 and 400 Ry for the plane-wave expansion of the wave functions and the charge density, respectively, and the 4×4×3 Monkhorst-Pack *k*-point grid for the Brillouin sampling. For the modelling of the surface of $SnSe_2$, we used a slab of three layers. For make calculations more realistic, we also considered the presence of the Se vacancies in the top layer. The formation of the single vacancy corresponds to $SnSe_{1.88}$ for the outermost surface layer.

Physisorption enthalpies were calculated by the standard formula:

$\Delta H_{phys} = [E_{host+mol} - (E_{host} + E_{mol})]$,

where E_{host} is the total energy of pristine surface, and E_{mol} is the energy of the single molecules of selected species in empty box. In the case of water adsorption, we only considered the gaseous phase. The chemisorption energy is defined as difference between the total energy of the system with adsorbed molecule and the total energy of same system after decomposition of the same molecule on the surface. For the case of physisorption, we also evaluated differential Gibbs free energy by the formula:

$\Delta G = \Delta H - T\Delta S$,

where T is the temperature and ΔS is the change of entropy of adsorbed molecule, which was estimated considering the gas→liquid transition by the standard formula:

$\Delta S = \Delta H_{vaporisation}/T$,

where $\Delta H_{vaporization}$ is the measured enthalpy of vaporization.

REFERENCES

1. Chung, K.-M.; Wamwangi, D.; Woda, M.; Wuttig, M.; Bensch, W., Investigation of SnSe, $SnSe_2$, and Sn_2Se_3 Alloys for Phase Change Memory Applications. *J. Appl. Phys.* **2008**, *103*, 083523.

2. Guo, C.; Tian, Z.; Xiao, Y.; Mi, Q.; Xue, J., Field-Effect Transistors of High-Mobility Few-Layer $SnSe_2$. *Appl. Phys. Lett.* **2016**, *109*, 203104.
3. Smith, A.; Meek, P.; Liang, W., Raman Scattering Studies of SnS_2 and $SnSe_2$. *J. Phys. C* **1977**, *10*, 1321.
4. Taube, A.; Łapińska, A.; Judek, J.; Zdrojek, M., Temperature Dependence of Raman Shifts in Layered $ReSe_2$ and $SnSe_2$ Semiconductor Nanosheets. *Appl. Phys. Lett.* **2015**, *107*, 013105.
5. Cox, P.; Egdell, R.; Flavell, W.; Helbig, R., Observation of Surface Optical Phonons on SnO_2(110). *Vacuum* **1983**, *33*, 835-838.
6. Lan, T.; Li, C. W.; Fultz, B., Phonon Anharmonicity of Rutile SnO_2 Studied by Raman Spectrometry and First Principles Calculations of the Kinematics of Phonon-Phonon Interactions. *Phys. Rev. B* **2012**, *86*, 134302.
7. Tanuma, S.; Powell, C. J.; Penn, D. R., Calculations of Electron Inelastic Mean Free Paths (Imfps). Iv. Evaluation of Calculated Imfps and of the Predictive Imfp Formula Tpp‐2 for Electron Energies between 50 and 2000 Ev. *Surface and interface analysis* **1993**, *20*, 77-89.
8. Cox, D. F.; Hoflund, G. B., An Electronic and Structural Interpretation of Tin Oxide Els Spectra. *Surface Science* **1985**, *151*, 202-220.
9. Giannozzi, P., et al., Quantum Espresso: A Modular and Open-Source Software Project for Quantum Simulations of Materials. *Journal of Physics: Condensed Matter* **2009**, *21*, 395502.
10. Perdew, J. P.; Burke, K.; Ernzerhof, M., Generalized Gradient Approximation Made Simple. *Physical Review Letters* **1996**, *77*, 3865-3868.
11. Barone, V.; Casarin, M.; Forrer, D.; Pavone, M.; Sambi, M.; Vittadini, A., Role and Effective Treatment of Dispersive Forces in Materials: Polyethylene and Graphite Crystals as Test Cases. *Journal of Computational Chemistry* **2009**, *30*, 934-939.
